# The Free Energy Principle and Free Markets

**DOI:** 10.3390/e28090956

**Published:** 2026-08-25

**Authors:** Karl Friston, Johan Medrano, Tim Verbelen

**Affiliations:** 1 Queen Square Institute of Neurology, University College London, London WC1N 3AR, UK; k.friston@ucl.ac.uk; 2 K. Lisa Yang ICoN Center, Massachusetts Institute of Technology, Cambridge, MA 02139, USA; 3 VERSES, Los Angeles, CA 90016, USA; tim.verbelen@verses.ai

**Keywords:** free energy principle, financial markets, active inference, portfolio management, Bayesian, risk, Helmholtz–Hodge decomposition, Laplace assumption

## Abstract

We apply the free energy principle to free markets by treating the Market as a random dynamical system with an attracting set, i.e., some characteristic states. This licenses a normal form for stochastic dynamics that inherits from the Helmholtz–Hodge decomposition. Equipped with this functional form—and a suitable parameterization—one can create a generative model of fluctuations in the value of assets and accompanying indicator variables. This affords the opportunity for prospective (*ex ante*) prediction, scenario modelling and forecasting that could, in principle, be applied to any complex dynamical system exhibiting stochastic chaos. Here, we illustrate the application to portfolio management—in the context of financial services—and use the (posterior) predictive densities over future paths to evaluate the expected free energy that underwrites active inference. In this application, active inference reduces to risk-sensitive control, which can be used to model the optimal decision-making of an agent or investor. In this setting, an investor is characterized by their prior preferences for a high rate of return under drawdown constraints. Using numerical studies and historical financial data, we quantify the improvement in portfolio management, relative to baseline policies.

## 1. Introduction

This paper introduces a generic approach to complex system modelling using variational procedures. These procedures are applied to financial modelling, which offers an interesting opportunity to combine generative modelling with optimal decision-making under uncertainty using active inference. What follows rests on two contributions. First, a way of modelling complex systems with both stochastic and chaotic aspects. Second, it shows how such modelling furnishes forecasts—in the form of posterior predictive densities—that can be used to reproduce Bayes-optimal decision-making. The latter contribution rests on modelling an ideal investor as an agent that conforms to the principles of active inference.

The kind of modelling described in this paper can, in principle, be applied to any complex system, e.g., from neuronal networks in clinical neuroscience—for epileptic seizure prediction—through to high-frequency trading in financial markets [1,2,3]. From the perspective of monetary and fiscal modelling [4,5,6], the requisite generative model is based on three assumptions:

- Assumption 1: The Market has characteristics states (i.e., there exists a nonequilibrium steady-state solution).
- Assumption 2: There are precise (aggregate) measures of fluctuations in the state of the Market and its assets
- Assumption 3: Assets have fair price equilibria that depend upon the state of the Market [7]

These particular assumptions were chosen to be as generic as possible, in relation to any complex system that restricts itself to some characteristic states. Assumptions 2 and 3 are specific to situations in which the observations or data are sufficiently large in number or dimensionality to license averaging. Similar situations present themselves in high-dimensional electrophysiology, with multiple channels, in which one can assume that the averaged data are effectively free of observation noise. The assumption of a fair price equilibrium is clearly specific to the free market; however, it is also generic in the sense that certain latent states are enslaved by other states. In other words, certain states generating data have dynamics that are contextualised by some other states (that change more slowly than the states they contextualise).

Together, these assumptions lead to a generalization of dynamic general equilibrium modelling [8,9] (DGE)—which covers macroeconomic methods used for policy analysis, forecasting and explaining historical timeseries data—to nonequilibrium or far-from-equilibrium dynamics. In this treatment, nonequilibrium dynamics are read in the technical sense of breaking detailed balance. In this setting, there is a fundamental difference between an equilibrium and nonequilibrium steady state. The nonequilibrium steady state necessarily involves itinerant dynamics (e.g., cycles, oscillations and chaotic orbits), such that there is a clear difference between running the system forward and backwards in time. In contrast, equilibrium steady states have point attractors and implicit time reversibility (i.e., the system looks the same under a time reversal protocol).

The generalization of DGE to nonequilibria rests upon Assumption 1: markets have characteristics states. This assumption places strong constraints on the stochastic dynamics that generate financial time series. In particular, the notion that the Market exists—in virtue of returning to states that are characteristic of a market—implies the existence of a pullback attractor, namely an attracting set of states to which the dynamics self-organize [10,11,12].

In physics, this implies the existence of a nonequilibrium steady-state (NESS) solution that—via the Helmholtz–Hodge decomposition—possesses dissipative and non-dissipative parts [13,14,15]. The non-dissipative parts are crucial for the current treatment, as nicely motivated in [16]. It is these non-dissipative, divergence-free or solenoidal components that underwrite business and market cycles at multiple scales [4,8,9]. Much of what follows rests upon this generalization, which furnishes an expressive but principled generative model of financial timeseries that is apt for forecasting and, implicitly, policy analysis and portfolio management.

The approach to portfolio management illustrated in this paper rests upon the physics of self-organization afforded by the free energy principle and its application to active inference as planning [17,18]. In brief, one can apply variational principles of least action to describe the behavior of agents in terms of minimizing a quantity known as expected free energy [19,20]. In the absence of any ambiguity, this reduces to risk-sensitive control in engineering and behavioral economics [9,21,22,23]. In what follows, we show how the modelling of random dynamical systems, under the Helmholtz–Hodge decomposition, affords predictive (posterior) densities over future states of the Market, and how these density dynamics can be used to select optimal policies via active inference.

This paper comprises four sections. The first considers the Market as a complex dynamical system—with certain characteristics—that lead to a normal form—based on the Helmholtz–Hodge decomposition—for subsequent state-space modelling. Crucially, this kind of dynamic causal modelling [24] can accommodate stochastic chaos and the admixture of endogenous and exogenous fluctuations that characterize financial markets [25,26,27].

Having established the normal form, Section 2 considers the use of standard variational model inversion procedures [28] to identify the system at hand, given some historical timeseries. We will use multiple indicator variables and a handful of aggregated exchange-traded funds (ETFs), namely an investment fund that holds assets—i.e., stocks, bonds or commodities—bought and sold on public stock exchanges. Having identified the parameters of the state-space model that best explains exemplar historical data, this section considers the ensuing characterization in terms of dynamical stability and the emergence of business cycles. This section concludes by asking a key question: is the Market chaotic? We address this by examining the Lyapunov exponents [29,30,31] associated with the dynamics, following system identification.

Section 3 turns to forecasting, namely evaluating the evolution of the probability density over market states and assets in the future. This section introduces a fast and efficient analytic solution—based upon a Laplace approximation to density dynamics [32,33,34]—and briefly addresses its face validity using solutions to stochastic differential equations used more commonly in numerical treatments (e.g., agent-based stochastic modelling [35]).

Having established the variational procedures required to estimate the predictive density over market states and assets in the future, Section 4 considers investing as active inference. This section introduces the central role of expected free energy in determining Bayes-optimal behavior, in the dual sense of optimal Bayesian design and Bayesian decision theory [36,37,38,39,40]. In the present context, the latter is emphasized through a reduction to risk-sensitive or path integral control [21,23], i.e., choosing the policy—e.g., rebalancing schedule—that minimizes the path integral of free energy expected under the predictive density above. We illustrate the putative optimality of this kind of policy optimization—using numerical simulations and legacy data—to see how an active inference investor performs in relation to buy and hold baselines, on the one hand, and an expected utility agent on the other. We close with a brief discussion of the limitations of this approach and potential generalizations.

## 2. Markets as Random Dynamical Systems

Our starting point for this treatment of free markets is the free energy principle [20]. In brief, the free energy principle is a variational principle of least action, due largely to *Feynman’s variational principle* that rests upon the Jensen–Feynman inequality [41,42,43]. The free energy principle describes the dynamics of any system that can be identified from a larger system within which it is contained. The requisite individuation turns upon a boundary or Markov blanket [44,45,46] comprising a set of states that render the internal states of the system conditionally independent of the external states, given the blanket states. This partition gives rise to a Bayesian mechanics [47] that shares exactly the same commitments as quantum, statistical and classical mechanics [20]. The only difference among these mechanics is that Bayesian mechanics pertains to how the internal states—of a particle, person or institution—stand in relation to external states. The technical details are not important here, other than to note that if any particle or institution—such as a free market—exists, then its flow (a.k.a. drift) can be expressed as the solution to its density dynamics, i.e., the solution that renders the change in probability density over states zero. The functional form of this solution is given by the fundamental theorem of vector calculus, namely the Helmholtz–Hodge decomposition [13,14,15]:

(1) x˙=f(x)+ωp˙(x)=0⇔f(x)=Q(x)∇ℑ(x)︸Conservative−Γ(x)∇ℑ(x)︸Dissipative−∇·Q(x)+∇·Γ(x)︸Correction term  ℑ(x)=−lnp(x)p(ω)=N(0,2Γ)

**Figure 1:**
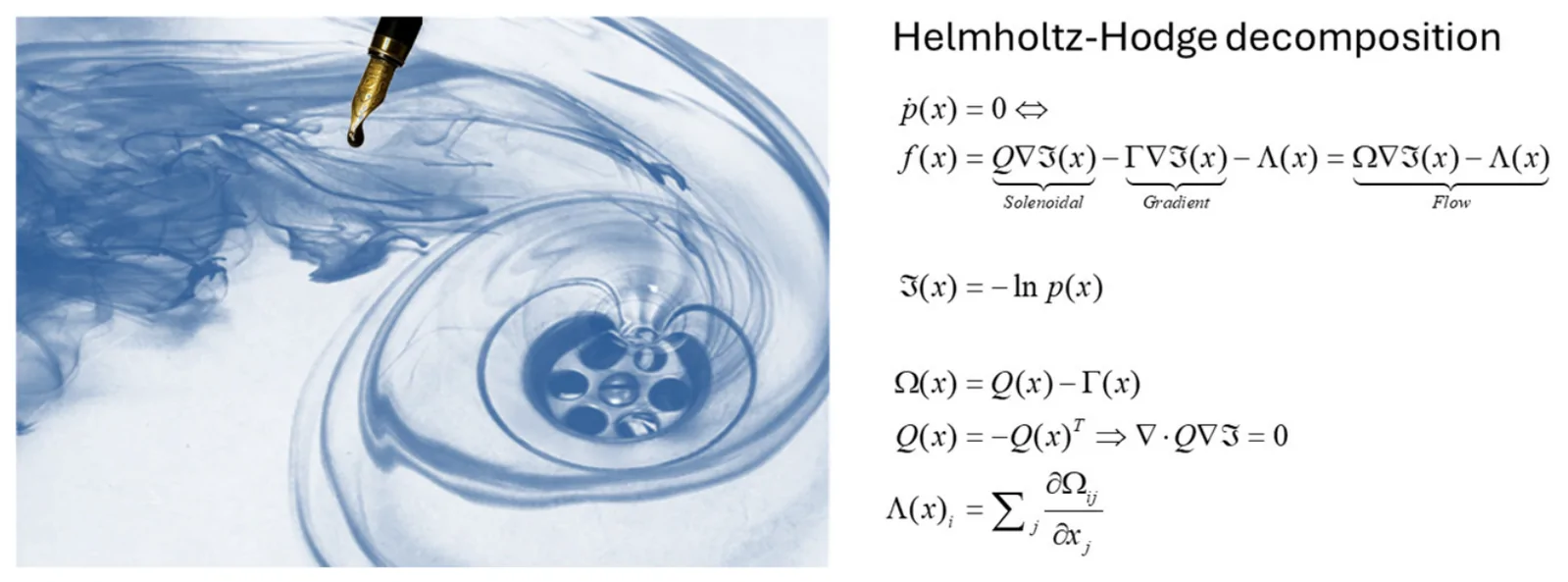
Solenoidal dynamics and the Helmholtz–Hodge decomposition. This figure provides equivalent formulations of the Helmholtz–Hodge decomposition, i.e., the functional form for the flow or dynamics of a system that has a nonequilibrium steady-state solution. Intuitively, the flow (i.e., drift) can be decomposed into solenoidal (i.e., divergence-free or non-dissipative) and gradient (i.e., curl-free or dissipative) components. These components are pictured on the left in terms of the swirling flow of water down a plughole. The final term is a correction (harmonic) term required under certain boundary conditions.

This normal form decomposes the flow into dissipative and non-dissipative (a.k.a. conservative) parts. The dissipative part flows down gradients of the negative log probability, known as *self-information* or *surprisal*, with state-dependent velocity parameterized by the symmetric matrix Γ(x). Conversely, the non-dissipative, curl or solenoidal part flows around the isocontours of surprisal, with state-dependent velocity parameterized by the antisymmetric matrix Q(x). Figure 1 illustrates this decomposition intuitively, with a picture of water flowing down a plughole. The downward flow corresponds to the dissipative gradient flow on the surprisal, while the solenoidal, circular flow corresponds to the non-dissipative or conservative part. Crucially, it is solenoidal flow that characterizes complex systems and, in particular, endows them with stochastic chaos and chaotic itinerancy [48,49,50,51,52]. For example, it underwrites oscillations in biotic self-organization, ranging from biorhythms through to lifecycles [53,54]. In the limit of very large systems—such as heavenly bodies—the random fluctuations are averaged away, leaving purely conservative or solenoidal dynamics [20]. Intermediate scales—that encompass living organisms through to free markets—evince characteristic cycles at multiple scales.

Technically, the prevalence of solenoidal dynamics in these large-scale systems breaks detailed balance and the time irreversibility that accompanies equilibrium physics [55,56,57] and DGE. The probability density over the states *p*(*x*) then becomes a nonequilibrium steady-state or NESS density [58,59,60,61]. From our perspective, the Helmholtz–Hodge decomposition furnishes a functional form for state-space modelling that gracefully accommodates business or market cycles [4,8,9], namely recurring fluctuations of prices in financial markets characterized by periods of growth (bull markets) and decline (bear markets). These cycles are generally thought to be engendered by investor behavior [62] and underlying economic factors, such as inflation, interest rates, corporate earnings, *et cetera*. The solenoidal—or divergence-free—component of the flow captures the endogenous, self-sustaining cyclicality inherent in markets, where resources are continuously reallocated without being dissipated. A classic macroeconomic example is the cyclical dynamic between employment and wage share (akin to the Goodwin model), where high employment increases labor’s bargaining power, raising wages and compressing corporate profits, which in turn dampen investment, leading to unemployment, which subsequently lowers wages, restores profits, and re-stimulates investment. Mathematically, this conservative, rotating reallocation of wealth between labor and capital manifests precisely as a solenoidal flow. In financial markets, this divergence-free dynamic is observable in sector rotation, where a conserved pool of total aggregate capital investment continuously circulates between asset classes (e.g., from growth equities during expansion, to defensive stocks during peaks, to bonds during contractions) depending on the economic season. Although economic thinking has moved towards the study of *economic fluctuations*—rather than business cycles *per se* [63]—we will refer to business and market cycles as an aspect of solenoidal flow, as distinct from random fluctuations.

This distinction touches on the debate as to whether the fluctuations of a business cycle are attributable to *exogenous* or *endogenous* shocks. Exogenous shocks are classically associated with stochastic fluctuations, while endogenous shocks are sometimes ascribed to deterministic chaos [64]. The neo-classical (and New Keynesian) view argues for exogenous causes, while the Keynesian school argues for endogenous causes. The current formulation encompasses both views through a model of *stochastic chaos*, in which fast random (exogenous) fluctuations coexist with—and engender—chaotic (endogenous) orbits due to the slow deterministic flow. In light of this, we will pay particular attention to the evidence for chaos in the ensuing models.

Crucially, (endogenous) solenoidal or cyclical phenomena often show a scale-invariant aspect, lasting for months or years in some instances yet also evident at faster timescales, usually with decreasing amplitude [65]. We will return to the separation of timescales implicit in solenoidal dynamics later because they afford key bounds on the predictability and frequency of trading.

From the perspective of the free energy principle, the states of the Market can never be observed directly by any market observer or investor. In other words, from a market actor’s perspective, all that can be seen or inferred about the states of the Market must be expressed in terms of indicator variables, asset prices, or other measurable quantities, while all influence on the Market must be expressed in terms of transactions and reallocation of wealth. These states mediating all interactions between an actor and the hidden market states constitute the Markov blanket of the actor or agent. Our agenda is to infer or estimate the states of the Market that are hidden from direct observation. However, although the hidden states are not directly accessible, we can model these states, given Assumption 1 and the ensuing functional form of their dynamics provided by the Helmholtz–Hodge decomposition.

## 3. Investors as Observers: State-Space Modelling

How does knowing the functional form of the hidden states of the Market help in financial modelling? In effect, we can install the corollaries of Assumption 1 in a state-space model, via the Helmholtz–Hodge decomposition. Consider a state-space model in which observable states are denoted by *y*(*t*) and hidden states by *x*(*t*).

(2) x˙=f(x)+ωy=g(x)+ε ω~N(0,2Γ)ε~N(0,γI), γ→0x~N(0,I)

The dynamics of the hidden states are known and decomposed into some lawful flow—given by (1)—plus some random fluctuations whose amplitude is given by 2Γ. In terms of economic theory, these can be regarded as endogenous and exogenous shocks, respectively. By Assumption 2, we can further assume that the observation noise *ε* is very small (because we are dealing with a large system and aggregated measures in which—by central limit theorem—random fluctuations are suppressed). However, the system is still inherently stochastic because the state noise can be arbitrarily large. If we make the simplifying (mild) assumption that there exists a mapping from some latent states to hidden states with a unit normal distribution at nonequilibrium steady-state, one can reduce this state-space model to a very simple form. From the distributional assumptions in (2), we have (here and throughout we use the dot notation to imply a dot or inner product: $A\cdot B=A^{T}B$):

(3) x˙=f(x)+ω  x=h(y)=s·(y−r·u(t))⇒y=g(x)=r·u(t)+s−1·x  E[x]=E[(y−r·u)]=0⇒r=y·u−∈ϑE[x·xT]=s·E[(y−r·u)·(y−r·u)]·s=I⇒s=E[(y−r·u)·(y−r·u)]∈ϑ

Here, we have taken a first order approximation to the mapping of *g*(*x*) from hidden states to observable states, whose inverse enables us to reduce the state-space model to the following nonlinear system, where hidden states are known, because *g*(*x*) is invertible. The inverse mapping *h*(*y*) can be regarded as simply detrending the timeseries and then whitening the data using the matrix square root of the ensuing sample covariance. The detrending uses temporal basis functions *u*(*t*); here, the first two components of a discrete cosine transform, modelling a time-invariant constant and a monotonic drift. Substituting (1) into (3) gives

(4) $$\dot{x}=Q(x)\nabla ℑ(x)-\Gamma (x)\nabla ℑ(x)-\Lambda (x)+\omega$$

All that remains is to parameterize the flow operators and surprisal in (4), such that we can estimate the unknown parameters. A generic parameterization would involve a polynomial expansion (in the states) of all the elements of the flow operators and surprisal

(5) Q(x,ϑ)=0…q1n·x⋮⋱⋮−q1n·x…0 lnΓ(x,ϑ)=g1·x…0⋮⋱⋮01n…gn·x  ℑ(x,ϑ)=12(x−p·x)·(x−p·x)  x=x1⊗x2⊗…⊗xnxi=[1,xi,12xi2,…,1m!xim]T  q={q11,q12,…,qnn}∈ϑg={g1,g2,…,gn}∈ϑp=[p1,p2,…,pn]∈ϑ

This parameterization has been explored in detail in the characterization of stochastic chaos using normal forms, such as the Lorenz system and coupled chaotic systems [50]. Under this construction the nonequilibrium steady-state density belongs to the exponential family, reducing to a unit normal distribution when the surprisal parameters tend to zero, as in (2):

(6) $$\begin{array}{cc} ℑ(x,\vartheta ) & =\frac{1}{2}(x-p\cdot x)\cdot (x-p\cdot x)\Rightarrow \\ \nabla ℑ & =(x-p\cdot x)\cdot (I-p\cdot \nabla x)\Rightarrow \\ \nabla^{2}ℑ & =(I-p\cdot \nabla x)\cdot (I-p\cdot \nabla x)+\ldots \\ & \Rightarrow \underset{p\to 0}{\lim}\;p(x)=N(0,I) \end{array}$$

For our purposes, a polynomial parameterization of the Helmholtz–Hodge decomposition furnishes a functional form for subsequent system identification. In this paper, we take a first-order approximation to the parameters of the flow operator (**q** and **g**) and the surprisal (**p**). Although the polynomial expansion is truncated at first order, the resulting system of equations is still highly nonlinear, but in a well-behaved way. For example, to ensure the dissipative part of the flow operator is positive definite, we take the exponential of a first-order expansion, which is, strictly speaking, infinite order in the states. Had we used a second-order approximation for **p** and **q**, the NESS density would have been fourth order in the states but still within the exponential family (i.e., a quartic form that is not unrelated to *q*-gaussian densities that arise from the maximization of Tsallis entropy). This means that, in principle, the nonequilibrium steady-state could have a super-Gaussian form, enabling the modelling of heavy-tailed probability densities over market states, e.g., [27].

However, by using a first-order polynomial approximation, we are implicitly using second-order approximation to surprisal. This means the NESS solution is Gaussian, i.e., a Laplace approximation. We will refer to this as the *Helmholtz–Laplace decomposition*. Note that although the steady-state solution is Gaussian, the nonlinear mapping to their flow in (4) renders the probability density over the motion (i.e., shocks) non-Gaussian. This follows because a substitution of variables renders a linear mapping under non-Gaussian assumptions equivalent to a nonlinear mapping under Gaussian assumptions, e.g., [66]. In this setting, exogenous shocks are modelled by random fluctuations, which take the flow of hidden states into regimes of high surprisal. In short, we have the functional form that is sufficiently constrained for system identification yet sufficiently expressive to model chaotic itinerancy in the Market—i.e., endogenous shocks—provided exogenous shocks can be absorbed into random fluctuations.

One might ask if this is a sufficiently constrained model for a free market? There is a further constraint that we need to consider that speaks to the nature of self-organization in general and Assumption 3—pertaining to fair price equilibria—in particular. Complex dynamical systems with a NESS solution always feature a separation of temporal scales into fast and slow [30,67,68,69,70]. Indeed, (1) expresses dynamics of hidden states in terms of a slow deterministic flow plus some fast fluctuations. These are designated as random because they dissipate before they can be observed and therefore can only be specified in terms of their sufficient statistics (e.g., covariance).

The same kind of separation can be applied to fluctuations under deterministic (endogenous) flow. Technically, these are the *paths of least action* that are pursued when the random fluctuations take their most likely value of zero. The implicit separation of timescales is usually assessed in terms of the Lyapunov exponents associated with the system’s (state-dependent) Jacobian. We will see examples of this later. At present, it is sufficient to appreciate that there are certain mixtures of states (i.e., eigenmodes) that decay very quickly and others that decay more slowly, as their eigenvalues approach zero from below. This is a generic aspect of any self-organization, as celebrated in the slaving principle of synergetics [30,70,71], through to center manifold theory [72]. In brief, any system can be decomposed into a small number of slow eigenmodes and a large number of fast eigenmodes that dissipate quickly. In synergetics, these correspond to *unstable* and *stable* modes, respectively.

In the context of financial modelling, this separation manifests as a distinction between (indicator) variables that reflect states of the Market that change slowly in relation to the faster fluctuations of other (asset) variables, which indicators contextualize. Assumption 3 comes into play if we consider that of the many factors that determine the price of an asset, much can be attributed to supply and demand and an appeal to the notion of a *fair price* equilibrium, e.g., [7,73]. Clearly, a fair price equilibrium will never actually be attained because the context established by the slow indicator variables will have changed before a fixed point equilibrium is reached. However, Assumption 3 means that hidden states generating asset prices are *enslaved* by hidden states of the Market that generate indicator variables. We can install the slaving principle [30,71] in the state-space model above by limiting state-dependent changes in surprisal—and implicit NESS density—to the asset states. Similarly, we can limit state-dependent changes in the amplitude of random fluctuations to the asset prices. In other words, the transformed indicator variables and asset prices become indicator and asset states that are distinguished by the fact that the nonequilibrium steady state of assets depends upon indicators but not *vice versa*.

In the absence of solenoidal flow (i.e., **q** → 0), the Helmholtz–Laplace model becomes a dynamic general *equilibrium* (DGE) model with state-dependent volatility (i.e., random fluctuations on the flow) and a fixed point equilibrium. A special case of this DGE would correspond to the assumptions that underlie the Black–Scholes model [74]. In short, the model in (4), with suitable prior constraints on the parameters—to separate indicator (enslaving) states from asset (enslaved) states—grandfathers general equilibrium models and conventional financial models. The outstanding issue is how to identify the model through posterior estimates of the requisite model parameters, i.e., polynomial coefficients in (5).

## 4. System Identification

This section addresses the problem of identifying the system at hand given historical timeseries. The illustrative data used here comprises a collection of (over 30) indicator variables and six representative aggregates of ETFs that includes one relatively risk-free asset (the first asset). Specifically, we assembled a dataset of financial and macroeconomic variables from 1 January 2008, to 20 November 2025. Equity and commodity prices (Table 1a) were sourced from Yahoo Finance using the yfinance API (https://ranaroussi.github.io/yfinance/reference/index.html accessed on 16 June 2026), while macroeconomic and financial series (Table 1b) were obtained from the Federal Reserve Economic Data (FRED) database via pandas_datareader (https://pandas-datareader.readthedocs.io/en/latest/ (accessed on 16 June 2026)).

These timeseries were decimated into weekly samples and log-transformed. The indicator variables were further reduced using the first three variates following a singular value decomposition of indicator and asset variables. This decomposition returns three linear mixtures that span the principal axes of state-space. The log-asset values were transformed into a rate of (log) return by evaluating the time derivative. Care was taken not to include future time points in this evaluation; in other words, the asset data comprised the average rate of log return over the preceding week.

**Figure 2:**
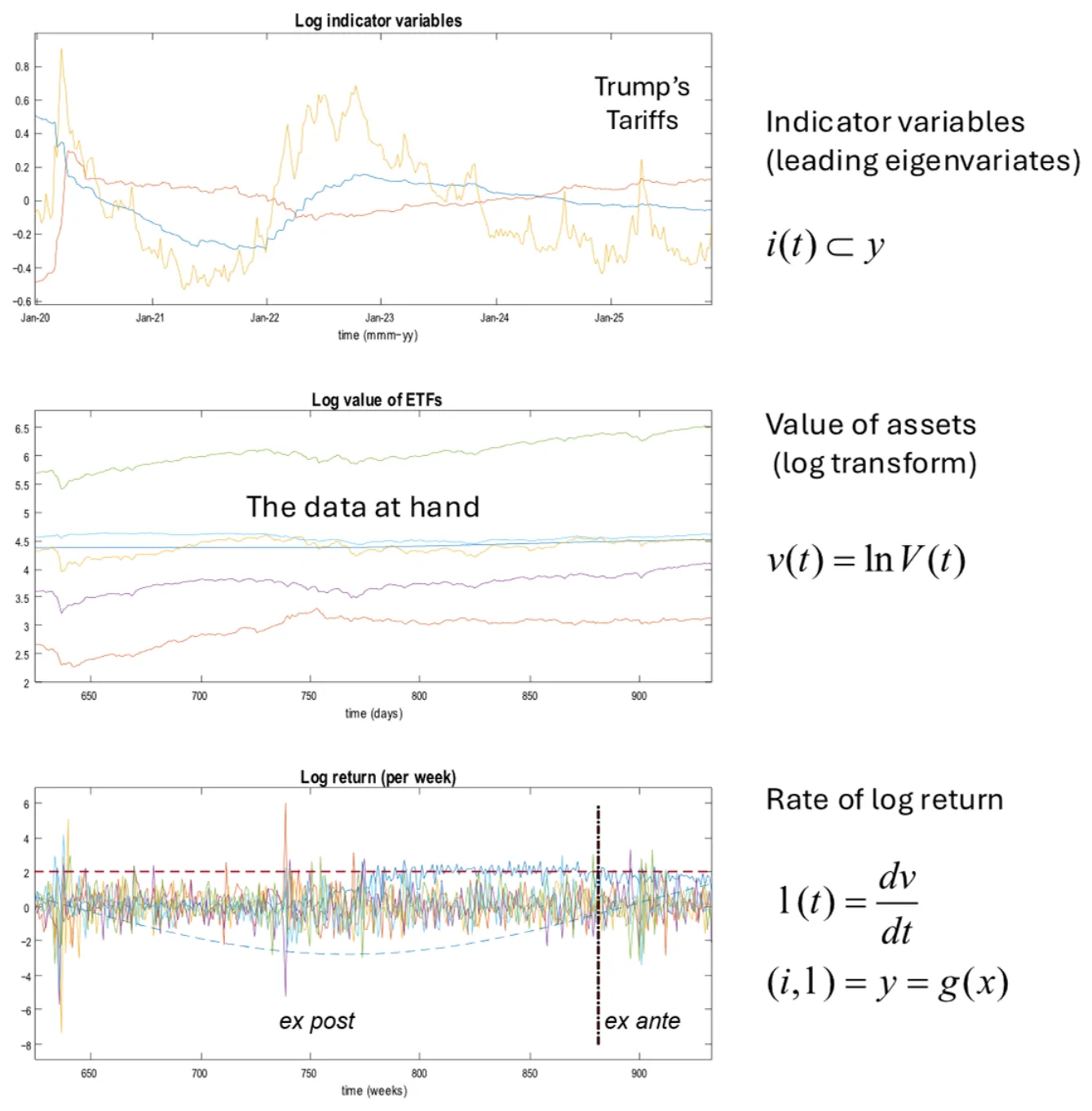
The data. This figure provides a graphical description of the data used for subsequent modelling. The (**upper panel**) shows the first three eigenvariates—of all the data at hand—as a function of time. These constitute (indicator) variables that reflect the state of the Market at any point in time. The (**second panel**) shows the corresponding value of assets (i.e., ETFs) following log transformation. The (**third panel**) plots the time derivative of log-transformed values, evaluated on a weekly basis. The dotted lines correspond to the constant and drift terms used to detrend the data. These are the first two components of a discrete cosine set for the period of interest: 256 weeks (*ex post*) and 52 weeks (*ex ante*) of forecasting.

The resulting timeseries y=(i,l) report the hidden states x=h(y) after detrending and whitening, as described in (3). See Figure 2.

Equipped with these data, one can now estimate the parameters of the Helmholtz–Laplace decomposition and thereby identify the system. This estimation problem reduces to system identification because the hidden states—and their temporal derivatives—are known quantities, by Assumption 2. This means the only unknown quantities are the parameters (**p**, **q**, and **g**) of the decomposition, which include the amplitude or log-variance of random fluctuations (**g**).

## 5. Variational Laplace

The requisite parameters can be identified in a straightforward way using standard variational procedures. In this instance, we used *Variational Laplace* [28,75] to provide posterior estimates of the model parameters. Variational Laplace (VL) is a generic scheme for inverting generative models under a mean field approximation, that is, factorizing unknowns, *ϑ*—e.g., hidden states and parameters—into a product of marginals [76,77]. In brief, VL uses a gradient descent on the variational free energy of an approximate posterior, which furnishes a lower bound on the log-evidence or marginal likelihood of the data, given a generative model, *m*. In machine learning, this is referred to as ELBO [77].

The efficiency and reproducibility of VL rest upon the Jensen–Feynman inequality that inherits from a fixed-form (Laplace) assumption about an approximate posterior and prior over unknowns. This assumption greatly simplifies the inversion. Formally, one can summarize VL as follows (following the convention in machine learning, variational free energy is maximised. In physics, the sign of variational free energy is reversed, and it is generally minimised. Later, we will use the physics version of expected free energy (whose path integral is minimised according to the principle of least action)):

(7) q(ϑ)=∏iq(ϑi) =argmaxqF  F=Eq[L]−Eq[lnq(ϑ)] =lnp(y|m)−DKL[q(ϑ)||p(ϑ|y)]≤lnp(y|m)L=lnp(y,ϑ)=lnp(y|ϑ)︸Likelihood+lnp(ϑ|m)︸Prior

The first equality expresses a mean field approximation, namely factorizing the approximate posterior over unknown variables into two or more marginal densities. The final equality expresses the generative model in terms of a log *likelihood* and *prior*. VL entails the iterative optimization of each marginal expectation until convergence, where the sufficient statistics of the *i*-th marginal are its mean and covariance:

(8) q(ϑi)=N(μi,Σi)Δμi=−∇ϑiϑiI(μi)−1·∇ϑiI(μi)  ∇ϑkiI(μi)=∇ϑkiL(μ)+12∑j≠itr(Σj·∇ϑjϑjϑkiL)∇ϑkiϑliI(μi)=∇ϑkiϑliL(μ)+12∑j≠itr(Σj·∇ϑjϑjϑkiϑliL)Σi=−∇ϑiϑiL(μ)−1  F=L(μ)+∑i12ln|Σi|

The key aspect of VL is that one only needs to optimize the expectations of the marginal posteriors. This is because the posterior covariances obtain analytically as a function of the expectations. For readers who want read about the variational basis of VL, please see [75]. For a tutorial, please see [28]. In our case, the generative model is specified by the Helmholtz–Laplace decomposition in (4):

(9) $$\begin{array}{cc} L & =\ln p(\dot{x},\vartheta )=\ln p(\dot{x}|\vartheta )+\ln p(\vartheta |m)\\ p\left(\dot{x}|\vartheta \right) & =N(f(x,\vartheta^{f}),2\Gamma (x,\vartheta^{\Gamma }))\\ p\left(\vartheta |m\right) & =N(0,\frac{1}{16}I)\\ f(x,\vartheta ) & =Q(x,\vartheta )\nabla ℑ(x,\vartheta )-\Gamma (x,\vartheta )\nabla ℑ(x,\vartheta )-\Lambda (x,\vartheta )\\ q(\vartheta ) & =q(\vartheta^{f})q(\vartheta^{\Gamma }),\;p,q,g\in \vartheta^{f},g\in \vartheta^{\Gamma } \end{array}$$

Usually, the approximate posterior is factorized into marginals over hidden states, parameters and variance components. However, here, we know the hidden states, which leaves a factorization over parameters and variance components that are optimized via fixed-point iteration according to (8). However, in the special case of (4), some parameters appear in both factors. This peculiarity of the Helmholtz–Laplace decomposition can be leveraged by noting that priors over the log-variances of the *i*-th random fluctuations are priors over the leading coefficient of **g***_i_* in the dissipative flow parameters. These priors can be specified as follows: if we know *a priori* that the motion of hidden states has both deterministic (endogenous) and stochastic (exogenous) contributions, one can specify the prior variance of random fluctuations as a proportion of the sample variance of the (known) time derivatives of the states. In what follows, we use ¼ of the sample variance of the *i*-th state:

(10) p(gi)=N(μi,Σi), μi=ln14Var(x˙i)00⋮, Σi=132    1    1    ⋱

In practice, shared parameters are initialized with the prior in (10), and the leading coefficients of $g\in \vartheta^{f}$ are replaced with the corresponding posterior estimate of $g\in \vartheta^{\Gamma }$. The remaining parameters are equipped with relatively uninformative priors with a mean of zero and a precision (i.e., inverse variance) of 16; see (9).

Given the generative model and historical data, we can now evaluate the approximate posterior over model parameters. Assumption 1 presupposes that there exists a nonequilibrium steady-state solution to the density dynamics. However, this solution will never be realized because the parameters of the flow can fluctuate very slowly. In other words, over the years, the contingencies and cause-effect structure of the Market will itself change [65]. This means that the historical timeseries—used for system identification—should be long enough to ensure efficient parameter estimation but short enough to ensure the parameters do not change over the period in question. Here, we use the preceding 256 weeks (i.e., about five years) to identify a model for predicting the subsequent 52 weeks (i.e., one year). See Figure 2. This period maximizes predictive accuracy, as assessed in the final section (i.e., evaluating annual performance under posterior predictive densities).

Before turning to forecasting and predictions, we address some key questions about the financial model. These questions can be about the dynamics entailed by model parameters or the prior assumptions that underwrite its structure. For example, what is the evidence for the enslaving of assets states? What is the evidence for state-dependent volatility? And so on. The answer to these kinds of questions rests on Bayesian model comparison [78,79], that is, comparing the evidence for models with and without a particular causal effect.

Bayesian model comparison turns on the fact that variational free energy is a bound approximation to log-evidence—from (7)—or marginal likelihood of the data, having marginalized over the unknown model parameters [75,77]. Furthermore, because we know the functional form of the posteriors, one can derive analytic expressions for the relative log evidence under different priors. This allows one to evaluate the change in log evidence analytically when removing various parameters by applying precise shrinkage priors (i.e., shrinking the parameter to a value of precisely zero). This is known as *Bayesian model reduction*; see [80] for a primer and [81] a fuller discussion. In short, Bayesian model reduction allows one to assess the evidence for a full model—with all parameters in play—against the evidence for a reduced model—with some parameters removed. This can be expressed in terms of a relative log-evidence or log-Bayes factor:

(11) $$\begin{array}{c} \ln p(y|m_{F})-\ln p(y|m_{R})=\ln\frac{p(y|m_{F})}{p(y|m_{R})}=\ln\frac{p(m_{F})}{p(m_{R})}\\ p(y|m_{R})=\int d\vartheta \underset{\mathrm{Posterior}}{\underset{︸}{p(\vartheta |y,m_{F})}}\underset{\mathrm{Evidence}}{\underset{︸}{p(y|m_{F})}}\underset{\mathrm{Priors}}{\underset{︸}{\frac{p(\vartheta |m_{R})}{p(\vartheta |m_{F})}}} \end{array}$$

The subscripts *F* and *R* denote full and reduced models, respectively. The final equality shows how the evidence for a reduced model can be computed analytically, given the posterior and evidence of the full model and model-specific priors. A difference in log evidence greater than five (natural units, or nats) is taken as very strong evidence in favor of one model over another [82].

Following model inversion, given the data in Figure 2, we compared the evidence for models with and without an enslaving of asset states, both in terms of their surprisal and volatility (i.e., amplitude of random fluctuations). The relative log evidence was 276.5 nats, providing extremely strong evidence for an influence of indicator states on asset states. When just removing state-dependent volatility, the log evidence fell by 17.3 nats, indicating very strong evidence for the enslaving of random fluctuations. These results can be read as evidential support for Assumption 3, which is the *raison d’être* for indicator variables in financial modelling. We now turn our attention from posteriors over models to posteriors over parameters to see what they tell us about the dynamics of market fluctuations.

## 6. Is the Market Chaotic?

The preceding sections have established a procedure for inverting a generative model of the Market that is apt to describe its density dynamics. In the remainder of this section, we consider the nature of this characterization from the perspective of dynamical systems theory and endogenous fluctuations. From a technical perspective, there are some important characteristics that can be derived from the system’s Jacobian at any point in time. The Jacobian is simply the rate of change of the flow with respect to hidden states and provides a compact summary of the causal power each state exerts over the others.

**Figure 3:**
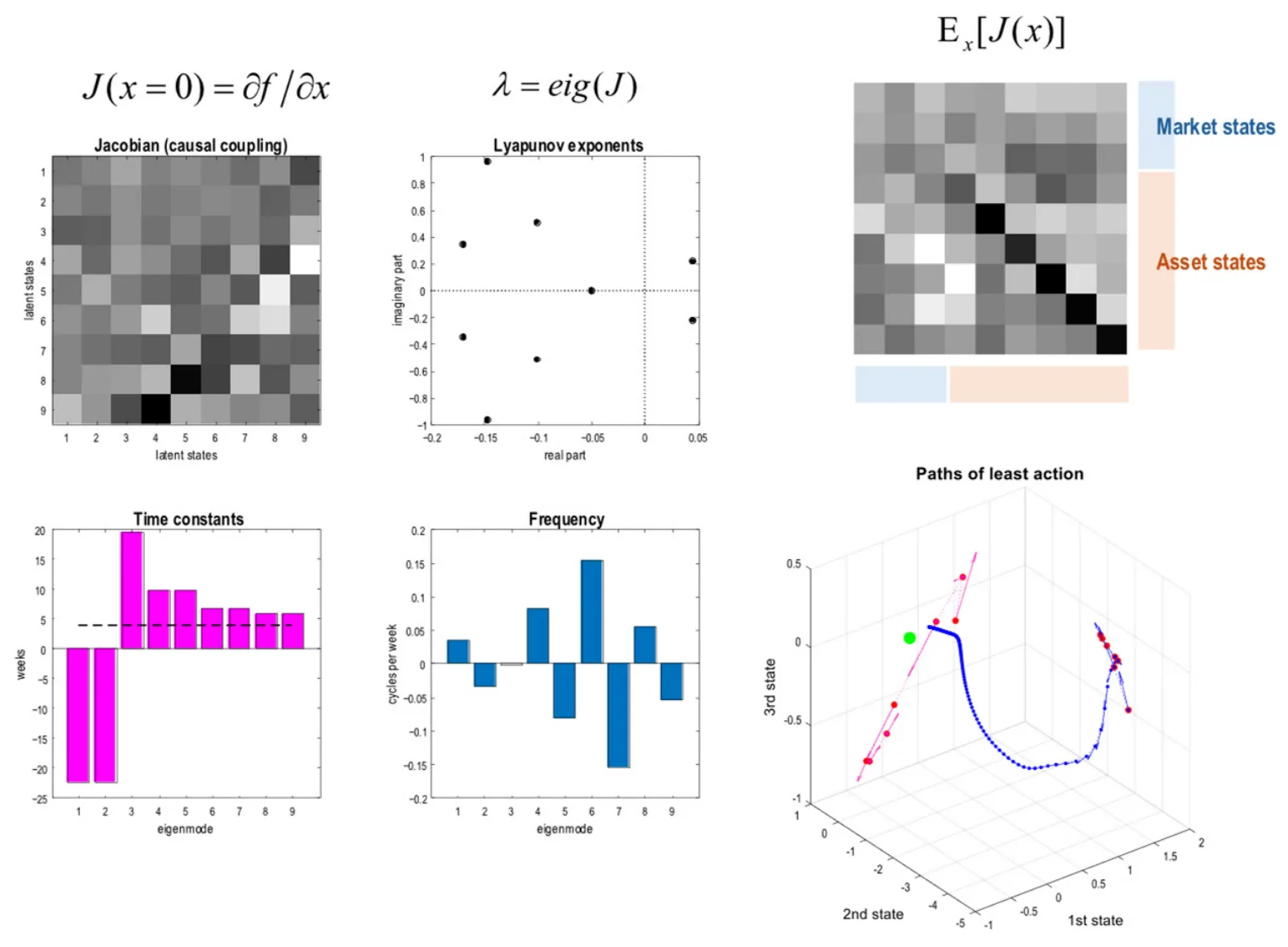
Flows and stochastic chaos. This figure reports various characterizations of the dynamics entailed by the system’s Jacobian. The four panels on the left depict the Jacobian—evaluated at the origin of state space—in image format (**upper left**), the corresponding eigenvalues (**upper right**), the inverse of the real parts of the eigenvalues or Lyapunov exponents (**lower left**), and the accompanying frequency that depends upon the imaginary part (**lower right**). The images have an arbitrary greyscale, with black denoting the (negative) minimum and white the (positive) maximum values. The panels on the right show the expected Jacobian evaluated numerically over state-space. The (**lower-right panel**) shows two arbitrary streamlines or paths of least action. The blue line corresponds to the path through the state-space of the first three indicator states, while the magenta lines report the equivalent paths for the first three asset states. Certain points on these paths evince an exponential divergence of trajectories (i.e., positive Lyapunov exponents), which are highlighted with red dots. The arrows correspond to the direction of flow at each time point upon the path.

Figure 3 shows the Jacobian evaluated at the most likely state of the Market, namely the origin, when all the states are at zero. The elements of the Jacobian can be read as the influence of one state on the flow of others, such that positive values indicate an increase and negative values a decrease. For example, the first indicator state (state 1) has a positive effect on the first asset state (state 4) but a negative influence over the second asset state (state 5). Note further that there is coupling among the asset states. In other words, if one asset state is high, it will variously increase or decrease the movement of other assets.

These influences can be characterized in terms of rate (or time) constants with the eigenvalues of the Jacobian [31,83]. Because we are dealing with a system that has solenoidal components—and therefore breaks detailed balance—the Jacobian has antisymmetric and symmetric components, parameterized by **q** and **g**, respectively. The antisymmetric components contribute to the imaginary parts of eigenvalues. These are shown in the complex number plane in the upper-middle panel. The real part of these eigenvalues, acquired from the symmetric components, can be read as the rate of decay of the corresponding eigenmodes (i.e., linear mixtures of states). In other words, some modes decay or dissipate very quickly and others more slowly, endowing the dynamics with multiple time constants. These characteristic time constants correspond to the negative inverse of the real parts. These are shown on the lower left. Interestingly, in this example, some of the time constants are negative. This reflects the exponential divergence of states (i.e., negative decay). This tells one immediately that the system at hand is chaotic, with local exponential divergence of paths in state-space that will ultimately find regions where they converge again (to ensure a nonequilibrium steady-state solution). The imaginary parts correspond to the frequency with which the eigenmodes oscillate while they are decaying. For example, the principal negative eigenvalue—that has a time constant of about four weeks—fluctuates with a frequency of about 0.05, namely with a cycle of about 20 weeks.

From a dynamical systems perspective, one can now answer the question: is the Market chaotic? The existence of positive real eigenvalues (under a nonequilibrium steady-state solution) allows one to answer in the affirmative. A more comprehensive analysis involves estimating the expectation of the real eigenvalues over state-space. These are called (global) *Lyapunov exponents*. In certain chaotic systems, state-dependent divergence and convergence of paths lend the attracting set a fractal (i.e., fractional dimensional) aspect. Although we are dealing with stochastic chaos, one can estimate of the fractal nature of the attractor—in the absence of random fluctuations—using the Kaplan–Yorke conjecture to estimate the Hausdorff dimension [84]:

(12) $$\begin{array}{cc} J(x) & =\nabla_{x}f(x)\\ \lambda (x) & =eig(J(x))\\ d_{H} & \approx j+\frac{\sum_{i=1}^{j}\lambda_{i}}{\left|\lambda_{j+1}\right|},\;\mathrm{where}\;\sum_{i=1}^{j}\lambda_{i}⩾0,\;\lambda_{i}\geq \lambda_{i-1}\;\mathrm{and}\;\lambda_{i}=\int p(x)\lambda {\left(x\right)}_{i}dx \end{array}$$

This scores the average tendency for exponential divergence of trajectories and the accompanying fractional dimension of the underlying attractor. In this instance, we estimated the expected Lyapunov exponents by evaluating the Jacobian at each point on the orbit taken by the historical data. This direct calculation highlights the utility of having a generative model and can be contrasted with numerical estimates of Lyapunov exponents in terms of exponential divergent of trajectories, which do not leverage the constraints afforded by priors of a generative model.

The calculated Hausdorff dimension was 2.85. This can be compared with the Hausdorff dimension for the Lorenz attractor of 2.43 [85]. A more intuitive comparison could be the self-similar surface of the human brain, which has a Hausdorff dimension of 2.80 ± 0.05 [86]. This characterization applies to the deterministic part of the flow, namely the paths of least action. Examples of these paths are provided in the lower-right panel of Figure 3. In short, the Market identified here can be said to exhibit stochastic chaos, where the flow *per se* evinces chaotic itinerancy [51,52]. So how does this relate to market cycles and predictability?

From a financial modelling perspective, the principal Lyapunov exponent will become important later because it furnishes both a lower and upper bound on the frequency of rebalancing, e.g., the waiting time between transactions, which itself is treated as a random variable in some settings [3]. Technically, the largest negative Lyapunov exponent sets a lower bound on the deterministic part of the flow, describing the rate at which the most unstable mode dissipates. This acts like a watershed that separates the slow deterministic drift from the fast random fluctuations, i.e., endogenous and exogenous fluctuations, respectively.

From the perspective of rebalancing a portfolio, this means the predictable or deterministic dynamics have to be predicated on timescales that elude fast (random) fluctuations. In this example, a portfolio manager should rebalance every month, *at most*. The complementary upper bound relates to the fact that certain unstable moments dissipate with a time constant of four weeks. Therefore, there are certain modes or patterns that cannot be predicted at a longer timescale. This suggests that rebalancing should be considered every month, *at least*. In short, the stability analysis—afforded by Lyapunov exponents—provides a principled constraint on the scheduling of portfolio rebalancing. In the final section, we will consider the behavior of investors who commit to rebalancing at a rate determined by the principal (global) Lyapunov exponent. This is almost invariably between three and four weeks.

**Figure 4:**
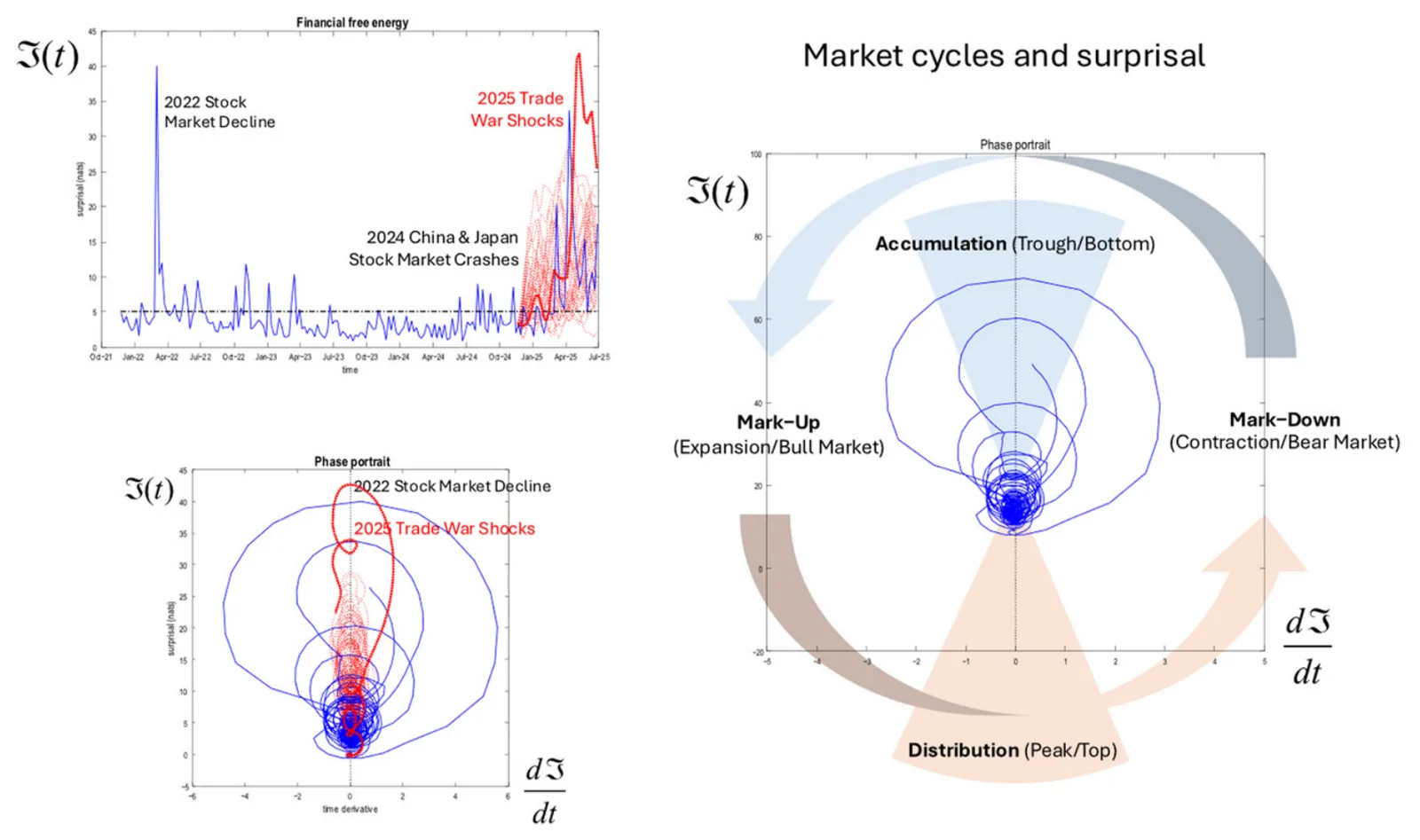
Market cycles and surprise. (**Upper-right panel**): This reports fluctuations in surprisal for the period covered by the data in Figure 2. The horizontal broken line corresponds to a surprisal of five natural units. The dotted lines are stochastic realizations (i.e., solutions) based upon parameter estimates using the preceding 256 weeks. Marked peaks—and clusters of peaks—are labelled with historical market shocks. (**Lower-left panel**): These data are presented as a phase portrait by plotting the surprisal against its rate of change. (**Right panel**): The phase portrait has been decorated with arrows depicting a business or market cycle that circulates through periods of distribution, markdown, accumulation and markup.

One might ask how the chaotic itinerancy above engenders business or market cycles. By construction, one can evaluate the surprisal of the Market at any point in time as an analytic function of the state of the Market using the maximum *a posteriori* estimate of the model parameters. This means one can track the state of the Market in terms of chaotic excursions into regimes of high surprisal. Figure 4 (upper-left panel) shows the timeseries of surprisal over the 256 weeks ending in July 2025. The peaks of surprisal correspond to various market shocks: the 2022 stock market decline, 2024 China and Japan stock market crashes and the 2025 trade war shocks are highlighted.

Market cycles can now be characterized by plotting the surprisal against the rate of change of surprisal, as in the lower-left panel. Again, by construction, this depicts fluctuations in surprisal as cycles through surprising and less surprising states of the Market, with occasional excursions into regimes of high surprisal. Figure 4 highlights the 2022 stock market decline and the 2025 trade war shocks. This characterization provides a mathematical image of market cycles, namely recurring patterns of price movement in financial markets with periods of growth and decline [4,8,9]. When expressed in terms of surprisal, they necessarily follow the four phases of accumulation, markup (expansion), distribution and lockdown (contraction). Notice that occasional large amplitude cycles are emitted in the context of faster, smaller cycles in regimes of relatively low surprisal.

Surprisal has a quantitative meaning in this setting. It is measured in natural units because surprisal is a negative natural logarithm of the probability of finding the Market in any particular state. In statistics, a log odds ratio of five or more is generally considered to be very strong evidence in favor of one model over another (in the sense of Bayesian model reduction above) [82]. We can interpret surprisal here in relation to the most likely state with a surprisal of zero; see (5). This means that a surprisal that exceeds five (dotted line in Figure 4) can be regarded as very strong evidence the Market has departed from its normal state of affairs. Note also the red dotted lines in Figure 4. These correspond to predictions of surprisal based upon stochastic solutions to the dynamics that form the basis of forecasting considered in the next section. One could argue that certain realizations anticipate the 2025 trade war, with marked increases in surprisal around March and April 2025. Indeed, one sample trajectory is remarkably similar to the fluctuations in surprisal that actually ensued (highlighted with a thick line). This does not mean to say that the model predicted the 2025 trade wars; rather, the fluctuations induced by Trump’s tariffs were consistent with the behavior of the Market over the preceding five years. We now consider analytic and numerical approaches to forecasting in more detail.

## 7. Forecasting the Future

Equipped with a generative model of the Market as a random dynamical system, one is now in a position to evaluate the probability density over states of the Market in the future. This provides the basis of *ex ante* or prospective forecasts and policy optimization. Mathematically, this rests upon evaluating the *posterior predictive density* over hidden states and ensuing fluctuations in asset prices. In many applications, this posterior predictive density is evaluated by solving stochastic differential equations and then using the resulting sample density over paths into the future. However, this can be a potentially expensive approach and produces different results with each sample.

A more efficient and reproducible evaluation of the predictive densities rest upon analytic procedures that, again, appeal to a Laplace approximation. In this instance, the Laplace approximation can be regarded as truncating the sufficient statistics of the predictive density to second order. Technically, this means converting the density dynamics entailed by the Fokker Planck equation into a set of deterministic differential equations governing the evolution of the moments of a Gaussian density (c.f., the method-of-moments). This can also be regarded as expressing the density dynamics in terms of the coefficients of polynomial basis functions of state-space and truncating to second order; c.f., [87]. The ensuing solution is relatively straightforward to implement, as follows.

One can exploit a fixed-form (Laplace) assumption about the predictive density by rewriting the density dynamics in terms of sufficient statistics, using an expansion of the flow around the expected state:

(13) $$f_{i}(x)=f_{i}(\mu )+\sum_{j}\frac{\partial f_{i}}{\partial x_{j}}x_{j}+\frac{1}{2}\sum_{jk}\frac{\partial^{2}f_{i}}{\partial x_{j}\partial x_{k}}x_{j}x_{k}+\ldots$$

Under Gaussian assumptions, we get

(14) μ˙i=fi(μ)+12∑jk∂2fi∂xj∂xkΣkjΣ˙ij=∑k∂fi∂xkΣjk+∑k∂fj∂xkΣik+Γij+Γji  Eq[x]=0, Eq[xixj]q=Σij

This can be expressed compactly in matrix form:

(15) μ˙i=fi(μ)+12tr(Σ·∇xxfi)Σ˙=∇xf·Σ+Σ·∇xf+2Γ  q(x(t))=N(μ(t),Σ(t))

The accompanying predictive posterior over indicator variables and assets in the future follows from (3) (i.e., under Assumption 2):

(16) $$\begin{array}{cc} q(y_{\tau }) & =q(i_{\tau },l_{\tau })=N(\mu_{\tau },\Sigma_{\tau })\\ \mu_{\tau } & =\nabla_{x}h\cdot \mu (\tau )\\ \Sigma_{\tau } & =\nabla_{x}h\cdot \Sigma (\tau )\cdot \nabla_{x}h \end{array}$$

For people interested in the mathematical details, please see [32]. For an illustrative application to medical timeseries analysis, please see [88]. The key thing to note about this Laplace approximation is that the mean of the predictive density depends upon the covariance, and *vice versa* (because the gradients and curvatures are evaluated at the current expectation). These equations are straightforward to integrate or solve, using local linearization [89], because we have a parameterized functional form for the flow, and we know the amplitude of the random fluctuations from the system identification above.

**Figure 5:**
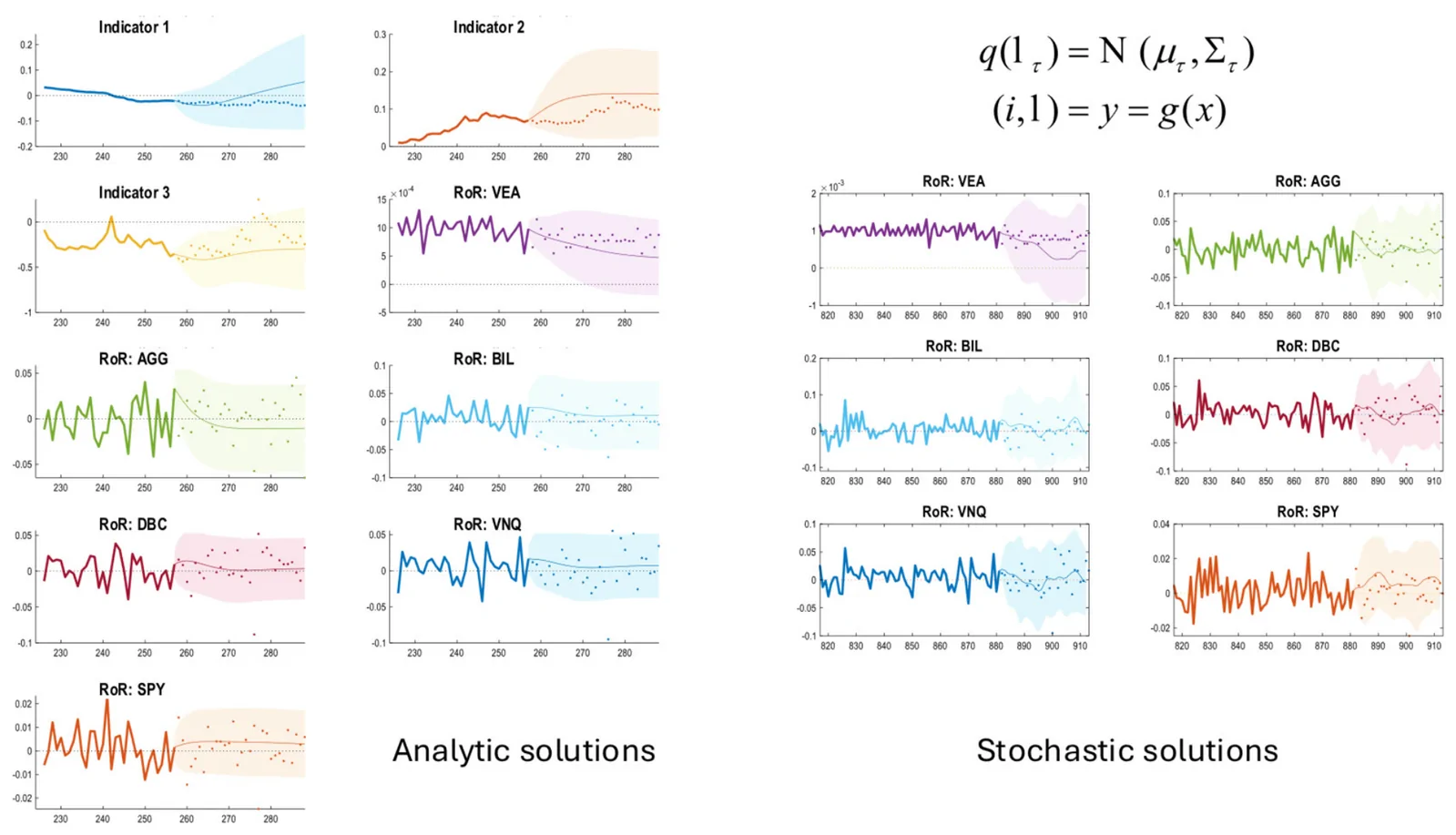
Predicting the future. The (**left panels**) depict the posterior predictive densities for the three indicator and six asset states. The shaded areas correspond to 90% Bayesian credible intervals, while the lines correspond to posterior predictive expectations. The dots correspond to the actual states observed following the onset of the predicted period, here, 32 weeks, starting at week 256. The (**right panels**) show the equivalent predictive densities for the six assets based upon a sample distribution of stochastic solutions for 32 paths into the future.

Figure 5 shows the analytic solutions to (15) for a period of 32 weeks, starting in late 2024 (at the same point the simulated trajectories of surprisal are shown in Figure 4). The predictive densities are shown in terms of their expectation and 90% Bayesian credible intervals. The dots correspond to the actual outcomes over this period. One would hope to see that 90% of the dots lie within the confidence intervals, which is largely the case. In this example, the initial density was set to be a Gaussian around the (known) hidden state at the beginning of the forecasting, with a covariance corresponding to the covariance of random fluctuations. This density then evolves towards its nonequilibrium steady-state solution. The rate of this evolution depends upon the amplitude of the random fluctuations by the second equality in (15). For example, the first state has a lower amplitude of random fluctuations because it is an indicator state, and therefore its variance increases more slowly than the last asset state, namely the ninth hidden state. Interestingly, the first asset state—a relatively risk-free state with a small volatility—is intermediate between the first and last market states.

One might ask whether a second-order approximation to the density dynamics is appropriate, given that the Helmholtz–Laplace decomposition has higher-order terms. One can establish the predictive validity of the analytic solution in relation to empirical samples based upon integrating the nonlinear stochastic differential equations upon which the Laplace approximation is based. These numerical solutions are shown in the right panel of Figure 5 and demonstrate a remarkable agreement with the analytic solutions. In this example, we used 32 solutions or trajectories in generalized coordinates of motion based upon an empirical estimate of their serial correlations. For people interested in the technical details of these numerical solutions, please see [90,91]. For an application to medical timeseries, please see [92].

In summary, we now have a set of standard variational procedures—based upon the Laplace approximation—that afford posterior predictive densities over future fluctuations in the rate of (log) return of various aggregated assets. In Section 8, we turn to policy optimization under these predictive densities using the principles of active inference.

## 8. Investors as Agents: Active Inference

In this section, we turn our attention to financial modelling from the perspective of a portfolio manager *who can act by* rebalancing their portfolio in a Bayes-optimal fashion. We start with an application of the free energy principle to the behavior of agents that are equipped with a generative model of the consequences of their action. Above, we considered the problem of inferring the hidden states of a market through their observable consequences, in terms of indicator variables and fluctuations in asset prices. Here, we promote this observer as an investor, i.e., an agent whose actions have observable consequences that can be forecasted using its predictive density.

Conceptually, this means augmenting observations with an observed return on investment (*r*) and supplementing the hidden states (*x*) with the wealth (*w*) apportioned among assets. Crucially, the agent enacts a policy (*π*) by transferring wealth from one asset to another. The asset and wealth states then determine the observed rate of (log) return *r*, which depends upon the (policy-dependent) wealth invested in each asset times their rate of (log) return *ℓ*:

(17) $$\begin{array}{cc} w_{t} & =\pi_{t}w_{t-1}\\ r_{t} & =l_{t}\cdot w_{t} \end{array}$$

Equipped with this action model, we can apply active inference to identify the optimal policy each time wealth is reinvested, that is, each time a portfolio is rebalanced. In what follows, the interval between rebalancing is four weeks—i.e., one month—in accord with the stability analysis of preceding sections.

Under the free energy principle, the states of the agent or observer pursue paths of least action, where action is the path integral of variational free energy used in the system identification of preceding sections; c.f., [61]. However, when applying the least action principle to planning and policy selection, the free energy functional becomes an *expected free energy* [17,20]. This follows because the outcomes—pursuant a particular policy—are random variables, in the sense that they have yet to be observed. In turn, this requires an expectation under the posterior predictive density, which was the focus of the previous section and provides the forecasted market states (16). Operationally, this means that one can pick the policy that minimizes (the path integral of) expected free energy G(π).

**Figure 6:**
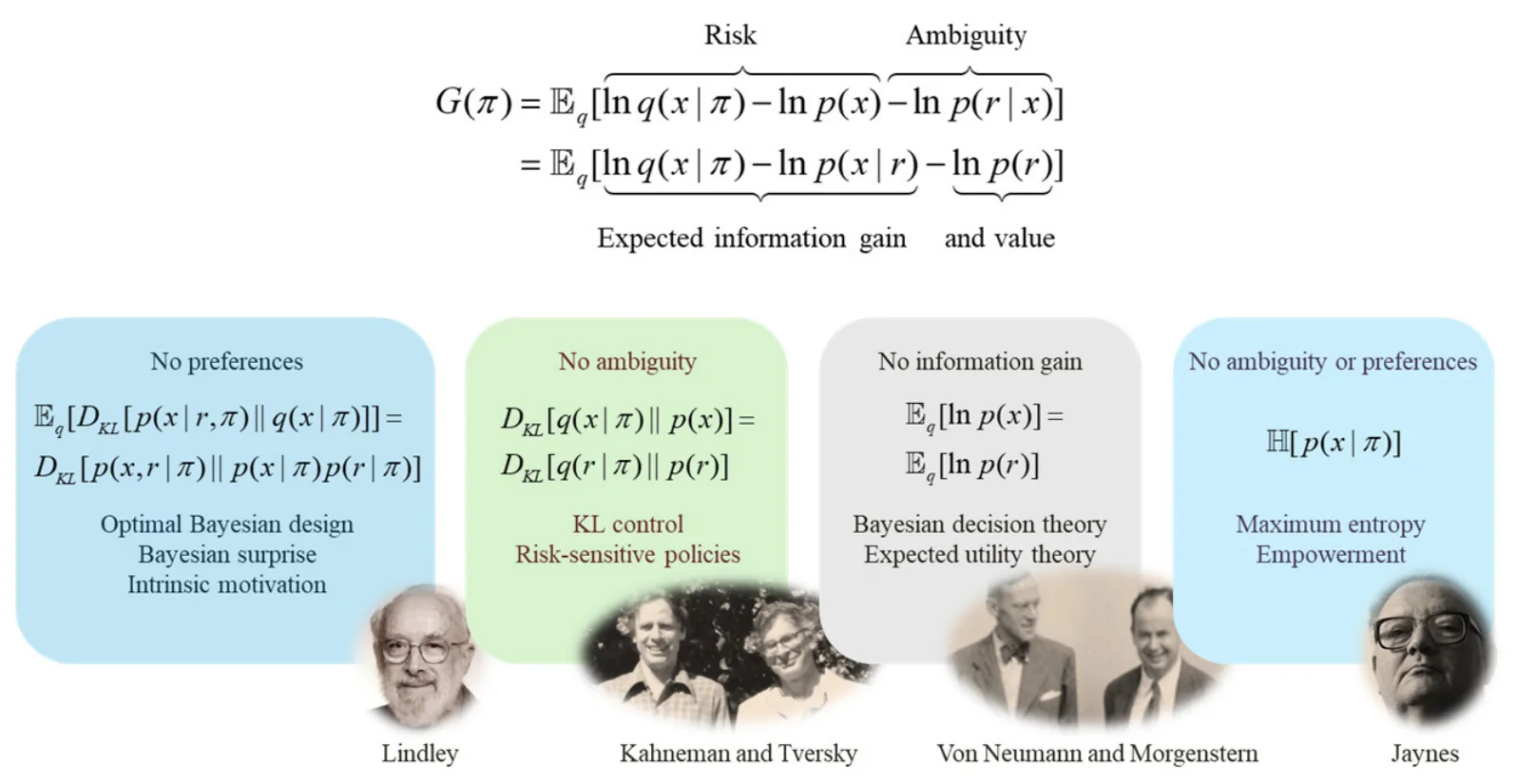
Active inference: the agent as investor. This figure illustrates the various ways in which expected free energy can be unpacked. Expected free energy subsumes several accounts in psychology, machine learning and economics. These special cases are disclosed when one removes particular sources of uncertainty. For example, if we ignore prior preferences or value, then the expected free energy reduces to expected information gain that underwrites optimum Bayesian design in statistics [39,40]—or intrinsic motivation in machine learning and robotics [94,95,96]. This is mathematically the same as expected Bayesian surprise and mutual information that constitutes salience in visual search [97,98]. Ignoring ambiguity leads to risk-sensitive policies in economics or path integral control in engineering [23,99]. Here, minimizing risk corresponds to aligning predictions to prior preferences. In the absence of expected information gain (i.e., reducible uncertainty), we are left with expected preferences or utility in economics—a construction that underwrites reinforcement learning and behavioral psychology. Maximizing expected utility under uncertainty leads to Bayesian decision theory [37,93]. Finally, if we consider an unambiguous world with uninformative preferences, expected free energy reduces to the negative entropy of posterior beliefs about the causes of data. This is a maximum entropy principle, proposed as a method of inference by Jaynes [100,101]. When conditioned on action, this corresponds to a form of empowerment [102,103], namely keeping options open.

Expected free energy subsumes the dual aspects of Bayes optimality, in the sense that it has information-seeking components—in accordance with the principles of optimum experimental design [39]—and utility seeking parts—in accordance with Bayesian decision theory under well-defined cost functions or constraints [37,93]. There are several ways of decomposing expected free energy; see Figure 6.

One can express (negative) expected free energy as *expected information gain* and *expected value*. Alternatively, it can be expressed in terms of *risk* and *ambiguity*. The risk in this instance corresponds to the KL divergence between anticipated and preferred outcomes, where the log-prior probability of—i.e., prior preference for—an outcome constitutes its utility or value. The ambiguity term is a conditional entropy over outcomes, conditioned upon hidden states. In the current application, we can ignore the ambiguity due to Assumption 2. This means that expected free energy reduces to the objective function used in path integral [99,104], risk-sensitive [21,23] or model-predictive [105] control.

In summary, we can use the predictive density afforded by system identification to evaluate the relative probability of various policies in terms of their expected free energy or, in this instance, risk. More precisely, we require the path integral of the risk expected under the posterior predictive density. Note that, in line with active inference, the approximate posterior optimises a variational bound on log evidence or marginal likelihood. In short, the approximate posterior minimises variational free energy, while policy selection minimises expected free energy, where expected free energy is evaluated under the approximate posterior.

The (path Integral of) expected free energy is generally evaluated for all plausible or allowable policies, and the most likely policy is selected for action. This policy is characteristic of the kind of agent in question, as specified by their prior preferences over outcomes. In the current application, we have a well-defined set of prior preferences that specify a good investor. This allows us to take the path integral of expected free energy—under the posterior predictive density in (16)—to identify the most likely policy a ‘good’ agent would adopt. Practically, this would constitute some recommendation, conditioned upon the specification of the agent’s prior preferences. In this example, we consider the prior preferences to be a Gaussian distribution over rate of (log) return that is substantively greater than zero, under the constraint that a negative rate of return is unlikely. The best of *k* policies at time *t* is then

(18) $$\begin{array}{cc} \pi_{t} & =\arg\min_{\pi }\sum_{\tau }G_{\tau }(\pi_{k})\\ G_{\tau }(\pi_{k}) & =D_{KL}[q(r_{\tau }|\pi_{k})||p(r)]-\underset{=0}{\underset{︸}{E_{q}[\ln p(r_{\tau }|l_{\tau },w_{t})]}}\\ p(r) & =N(\mu_{p},\Sigma_{p}),\;s.t.\;\int_{-\infty }^{0}p(r)=0.01\\ q\left(r_{\tau }|\pi_{k}\right) & =N(\mu_{q},\Sigma_{q}),\mu_{q}=\mu_{\tau }\cdot w_{k},\Sigma_{q}=w_{k}\cdot \Sigma_{\tau }\cdot w_{k},w_{k}=\pi_{k}w_{t}\\ q(l_{\tau }) & =N(\mu_{\tau },\Sigma_{\tau })\; \end{array}$$

This specification of prior preferences corresponds to an investor who expects, *a priori*, outcomes with a Sharpe ratio [106] of about 2.3 and a high rate of return. The Sharpe ratio measures risk-adjusted performance in terms of excess return per unit of volatility (i.e., standard deviation):

(19) $$\rho =\frac{\mu_{p}-\mu_{0}}{\sqrt{\Sigma_{p}}}$$

Here, $\mu_{0}$ is the expected risk-free rate of return, and $\mu_{p}$ is the preferred or expected rate of return. We consider a high (annualized) return to be 256%. This corresponds to the upper range achievable in practice (i.e., about two or three standard deviations above a rate of return of zero). Naturally, these priors are unlikely achievable but rather aim to provide the agent with an optimistic. This insufflates a desire to achieve as high returns as possible and evinces choice behaviour characteristic of an optimistic but risk-sensitive investor. We will see later that even though the Market will not admit the kinds of returns the investor prefers, through minimising risk, the divergence between optimistic preferences and actual outcomes is minimised. The choice of the expected return and Sharpe ratio is fairly arbitrary here. One could use the current formalism to estimate the prior preferences implied by an investor’s choices in the spirit of computational phenotyping. In other words, the prior preferences simply characterise the kind of investor or agent at hand.

Having specified prior preferences and the functional form of expected free energy, the only outstanding issue is to specify the policies from which to select. Here, we specify a policy as the composition of costly transfers:

(20) πk=Tij·Tuv·Tpq T11=T22=…=1    1    ⋱    1, T12=1Δ−c   1−Δ    ⋱    1, …

Here, each transfer redistributes a fixed proportion of wealth (Δ) from one asset to another with transaction cost *c*. Three transfers can be composed in any order to provide a flexible repertoire of policies, ranging from doing nothing (*π*_1_ = *T*_11_ · *T*_11_ · *T*_11_) through to moving ~Δ^3^ from one asset to another. Here, we use Δ = 50% such that the agent can move at most about 88% of wealth from one asset to another at every rebalancing. This particular set of policies is extremely flexible (with 46,656 alternatives) but precludes the movement of all wealth to one asset at any one time. The expected free energy of these policies can be evaluated quickly and efficiently, within about a second on consumer-grade hardware.

**Figure 7:**
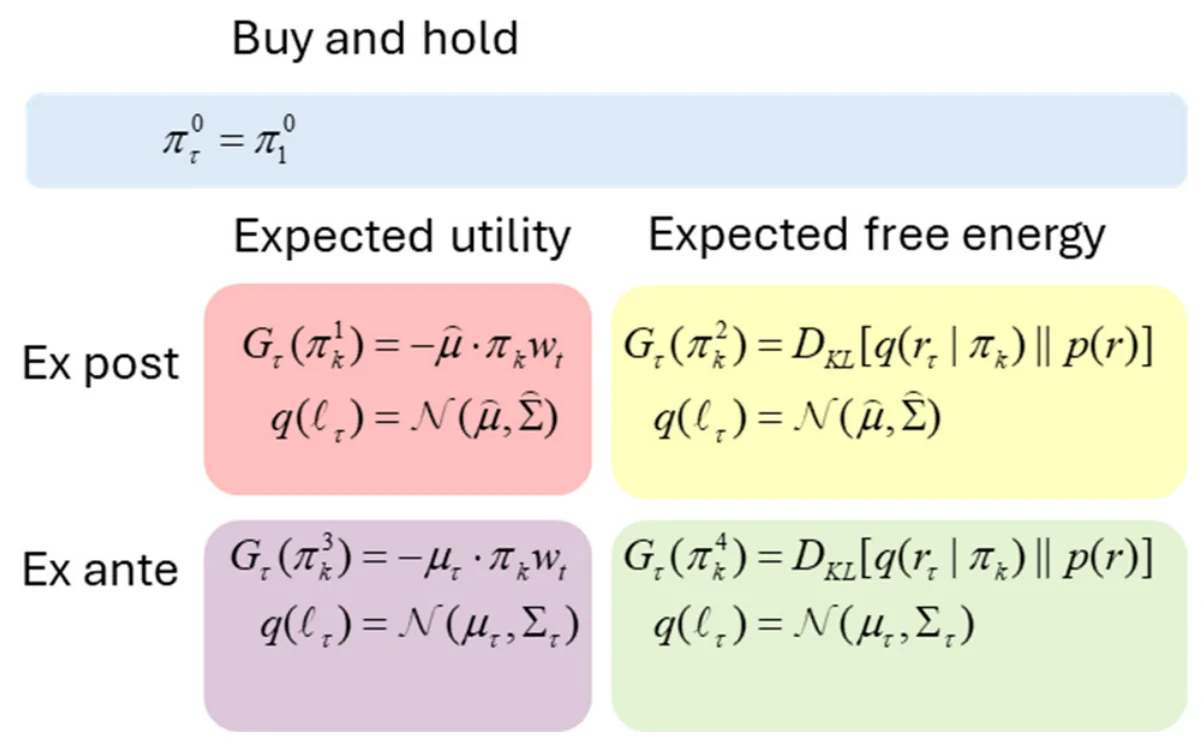
Alternative investment strategies. This schematic depicts the four investment strategies considered in the numerical studies, in relation to a baseline buy and hold policy. The four strategies constitute a factorial design. The first factor is whether the predictive density is based upon prospective (*ex ante*) posterior predictive densities or retrospective (*ex post*) predictions based upon the sample density over the preceding 16 weeks. The second factor pertains to the strategy, based upon expected free energy—which picks the policy that minimizes the expected complexity or risk—versus a simple expected utility investor, who picks the policy that maximizes predicted rate of (log) return. The bars denote *ex post* sample estimates of the mean and covariance of the rate of return for each of the six assets.

Having specified allowable policies—and the objective function for policy optimization—one can now simulate the behavior of a good agent. For comparative purposes, we considered five kinds of policies. We will refer to these as *investment strategies*; see Figure 7. These investment strategies were illustrative of the relative contribution of different modelling assumptions. The first was a simple *buy and hold* strategy, starting with an initial investment at the beginning of each year, reflecting a reasonably balanced portfolio (i.e., equally apportioned wealth to each of the six assets). This buy and hold strategy can be seen as the limiting case of an optimal policy in which the interval between rebalancing increases from four weeks to one year. We then considered four further strategies that were distinguished in terms of whether policies were selected (i) on the basis of *expected free energy* versus *expected utility* and (ii) whether the predictive densities were based upon the *ex ante* predictions above or using *ex post* predictions based upon the preceding 16 weeks of asset prices, in other words, using prospective (*ex post*) predictions of fluctuating prices or simply assuming (*ex ante*) that assets would continue to behave as they did in the preceding 16 weeks, with a constant mean and covariance.

The following numerical studies are not presented to illustrate a quantitative advantage of the current application of active inference. Rather, they are presented in order assess the relative contribution of different modelling assumptions. Specifically, this balanced factorial design allowed us to compare the relative contributions of the predictive modelling by comparing the *ex ante* with the *ex post* strategies, investigating the effect of an informed predictive density. In a complementary fashion, we were able to compare risk-sensitive against expected utility strategies under *ex post* and *ex ante* predictions, highlighting the role of accounting for uncertainty implicit in the use of expected free energy. Interestingly, this treatment highlights the fact that quantities like the (prior) Sharpe ratio have a dual role, specifying the intentions of an investor, through prior preferences, and scoring the engagement of the investor with the Market in terms of outcomes, i.e., a realised (posterior) Sharpe ratio. Note a small technical detail here: the *ex post* predictions were informed by the modelling because we used an exponential average of recent rates of return based upon the principal Lyapunov exponent afforded by the predictive modelling. The covariance was based upon the preceding 16 weeks. These empirical estimates are denoted with a bar over the mean and covariance in Figure 7.

**Figure 8:**
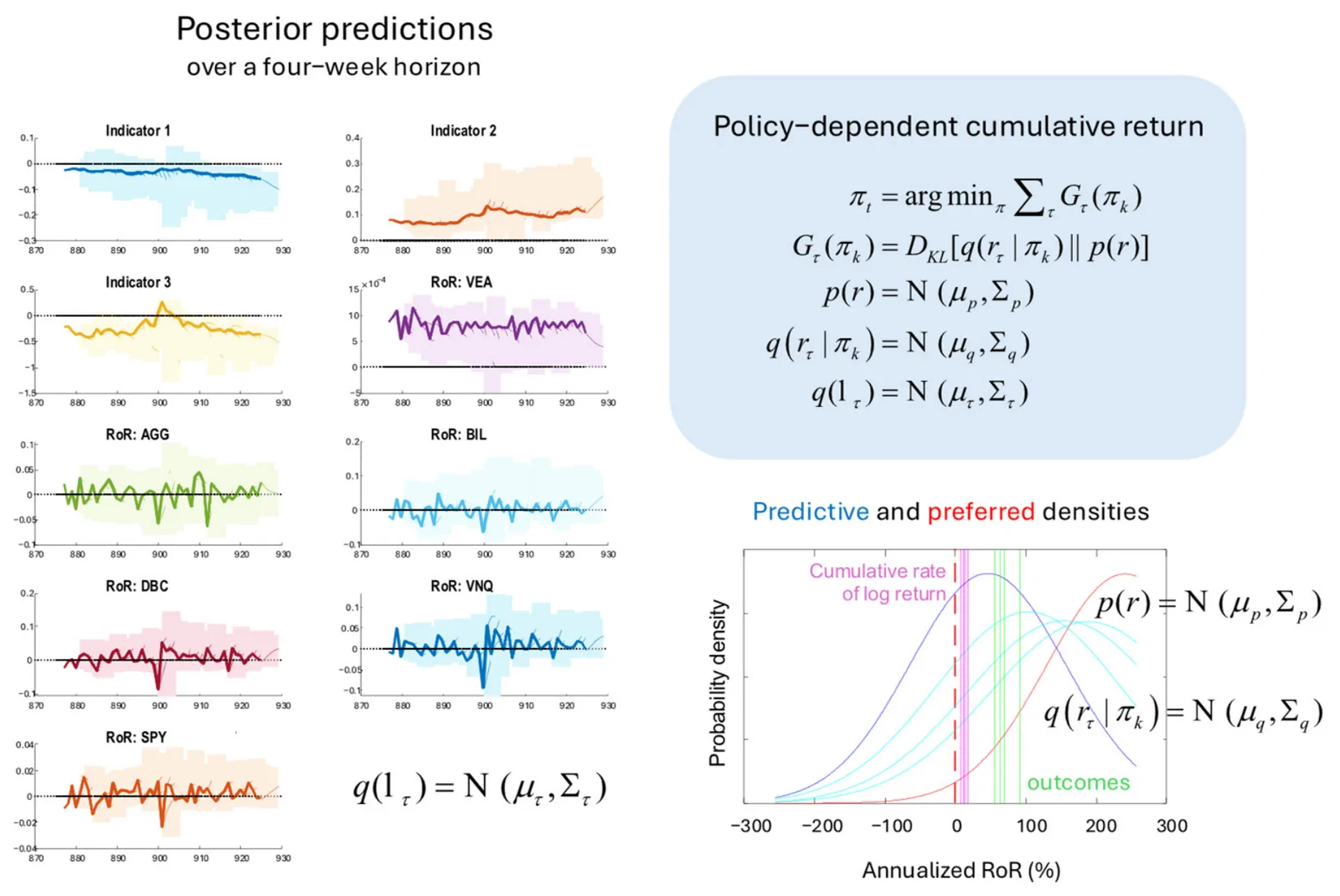
Predictions and policies. (**Left panels**): These show the succession of predictive densities following the format of Figure 5. Here, the densities are superimposed upon each other, after re-evaluation every four weeks, by each of four teams rebalancing one week apart. (**Right panels**): The (**upper panel**) summarizes the predictive density and ensuing risk used to identify the optimal policy under the KL strategies in Figure 7. The (**lower-right panel**) illustrates the preferred prior density over rate of return (red) and the closest attainable density, under the optimum policy, for the week ahead (blue line) and subsequent three weeks (cyan lines). The green lines correspond to the actual rate of return obtained over the ensuing four weeks. The magenta lines show the cumulative rate of return at the point these densities were evaluated. The vertical dashed line indicates zero rate of return.

Figure 8 shows the results of an exemplar simulation of portfolio management from November 2024 to October 2025, based upon parameter estimation from the preceding 256 weeks (i.e., about five years). The right panel shows the series of predictive posteriors over the indicator variables and assets in terms of 90% Bayesian credible intervals. The solid lines correspond to the posterior expectations, while the heavy lines show the actual outcomes. These posterior predictions cover four weeks into the future and are re-evaluated every four weeks, given the initial conditions afforded by data over the preceding week. In other words, they represent sequential forecasts of the sort shown in Figure 7. As above, one would hope that the actual outcomes lie within the credible intervals, which is the case. To mitigate against seasonality effects, we repeated these simulations four times, staggered by one week. Intuitively, one can think of this as having four portfolio management teams working independently using the same prior preferences and data, but only one team rebalances in any one week.

The right panels of Figure 8 show a particular instance of the predictive posteriors over outcomes and prior preferences (in blue or cyan and red, respectively). In this example, the preferred outcome was an expected rate of return of 256%, while the closest attainable (predictive) density had a mean of about 50% on the ensuing week but then subsequently increased over the month to about 180%. The actual outcomes over this period are shown in green. These outcomes move the cumulative rate of (log) return (in magenta) away from zero for each of the four weeks of the forecast.

**Figure 9:**
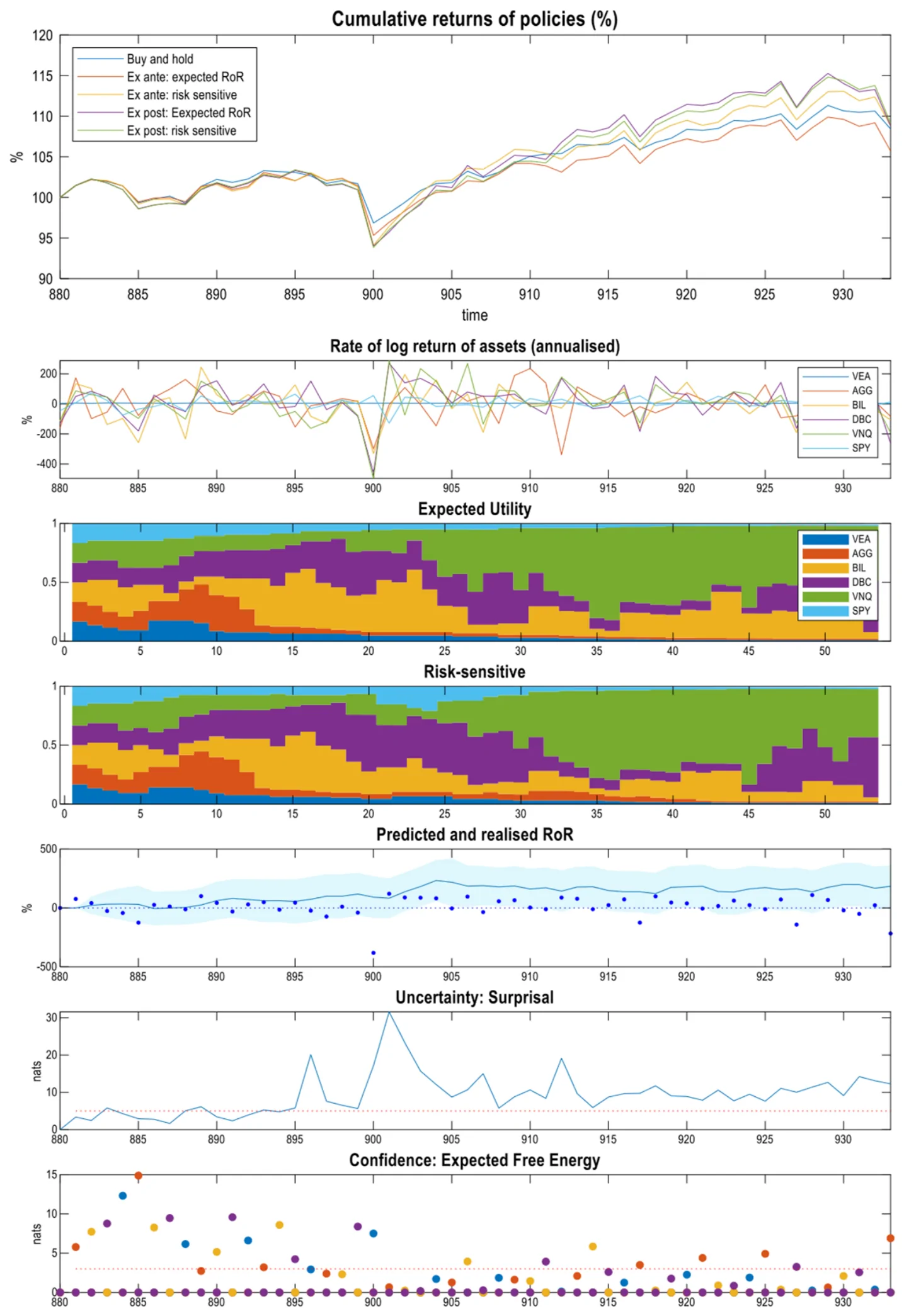
Predictions and performance. (**First panel**): This shows the cumulative rate of return of the five investment strategies listed in the legend. (**Second panel**): The underlying fluctuations in the rate of (log) return, expressed as annualized percentages. The (**middle panels**) report the rebalancing of wealth among the six assets for the expected utility (**upper panel**) and risk-sensitive (**lower panel**) investors, respectively. The (**fifth panel**) plots the posterior predictive density over rate of return in terms of 90% Bayesian credible intervals (shaded area) and expectations (blue line). The dots correspond to the actual outcomes. The (**sixth panel**) shows the time course of surprisal under the Helmholtz–Laplace model. In this example, the peaks—emerging at 895 and 900 weeks—correspond to the 2025 trade wars initiated by Trump’s tariffs. The (**lower panel**) reports the confidence placed in policy selection, as scored by the expected free energy: a higher value corresponds to a greater inferred risk for each of the four teams (shown in different colors). The horizontal line corresponds to a threshold of three natural units.

The resulting performance under the five strategies is summarized in Table 2 and Figure 9. Performance was assessed in terms of annualized rate of return, volatility and the ensuing Sharpe ratio (assuming a risk-free rate of return of 2%). In addition, we recorded the maximum drawdown. The five strategies performed as follows:

In this example, the risk-sensitive investor—equipped with *ex ante* predictive posteriors—was that the best performing, both in terms of the Sharpe ratio and annualized rate of return. This is reflected graphically in Figure 9 (upper panel). Quantitatively, the Bayes-optimal policy (*ex ante KL*, green line in the upper panel) obtained the highest rate of return and Sharpe ratio. Interestingly, the buy and hold baseline outperformed the *ex post*, expected utility investor, reflecting the danger of incurring too many transaction costs without forecasts with sufficient predictive validity. Figure 9 reports the predictions and performance over the year in question.

The top panel of Figure 9 tracks the cumulative returns—over a one-year period (November 2024 to October 2025)—averaged over the four portfolio management teams (each rebalancing every month). The underlying rate of (log) return of the six assets is shown in the second panel, and the proportion of wealth attributed to each of these assets is shown in image format in the middle panels. These panels report the changes in positions held for the expected utility and risk-sensitive strategies, using predictive posteriors, in the upper and lower panels, respectively. The lower-three panels report the predicted annualized rate of return as the expected value and 90% Bayesian credible intervals, where the red dots correspond to the actual or realized rate of return. The lower panels report the uncertainty about the state of the Market as scored by the surprisal under the Helmholtz–Laplace model. The lower panel reports the expected free energy for each of the teams (in different colors). The dotted red lines in the lower panels indicate when things are more surprising, defined operationally as surprisal or expected free energy exceeding five natural units.

At week 900 (in May 2025), the high levels of surprisal reflect the response to Trump’s tariffs and ensuing trade wars. At this point, all five investors experienced a substantive drawdown. Following this, all investors recovered in terms of cumulative returns. The differential recovery makes a key point: although market shocks cannot be predicted—because they are induced by random fluctuations—the systematic (endogenous) recovery from such (exogenous) shocks follows a relatively lawful and predictable pattern. If the accompanying *ex ante* predictions inform policy selection, the recovery from such shocks is accelerated in relation to the buy and hold baseline. The rebalancing under *ex ante* expected utility and active inference strategies is somewhat similar but differs slightly in terms of their commitment to different assets. Interestingly, in this example, the best performing strategy (*ex ante* KL) owes much to its investment in the green asset (VNC) at the expense of others, e.g., the cyan (SPY) asset. Note finally that the realized rate of returns (red dots) lies comfortably within the 90% credible intervals; however, they are generally lower than expected *a priori*. This reflects the fact that prior preferences were optimistic in relation to what the Market admits. This is a ubiquitous and Bayes-optimal aspect of active inference, sometimes read as an optimism bias [107,108,109,110] that underwrites optimal decision-making under uncertainty.

**Figure 10:**
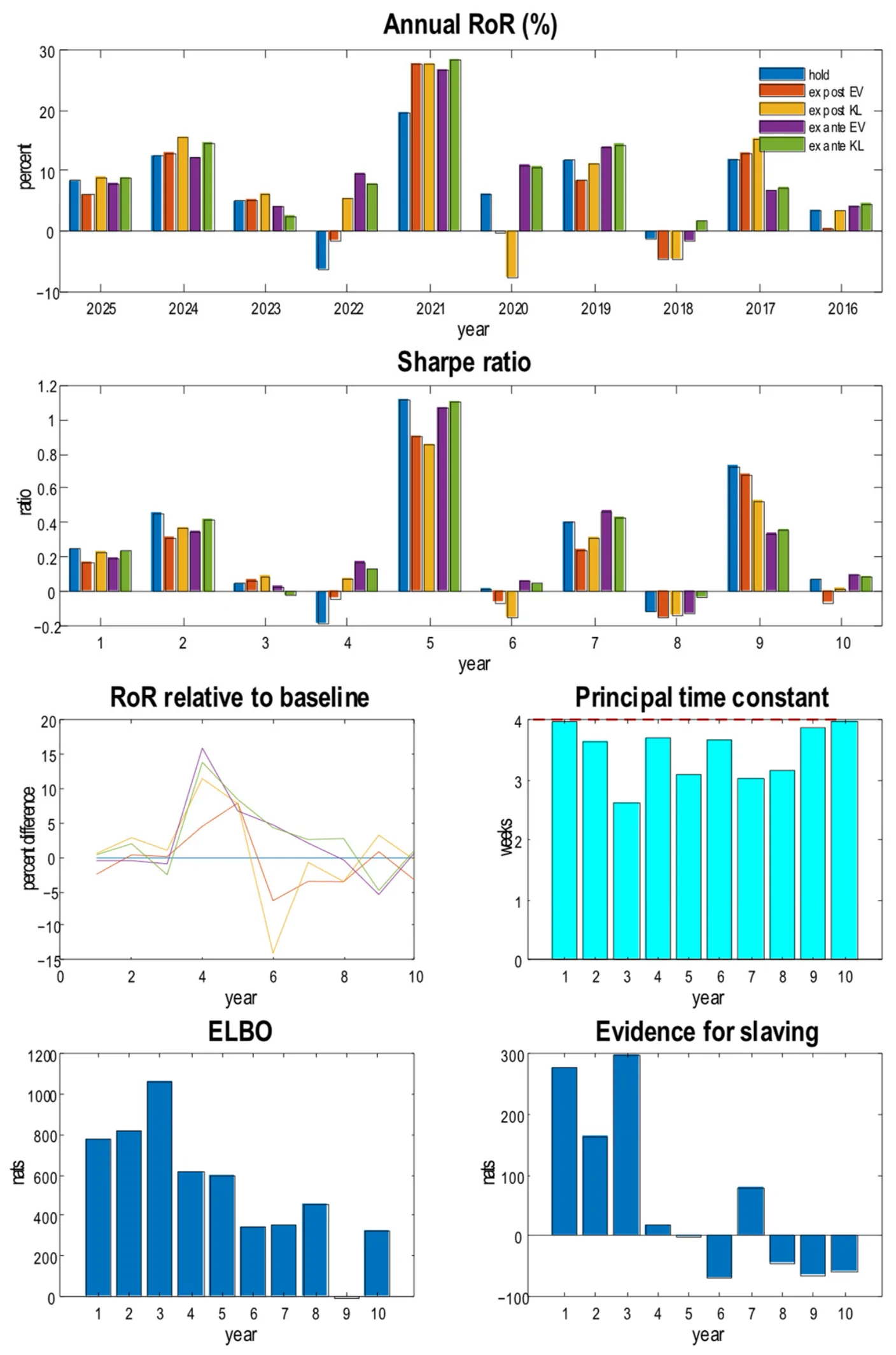
Ten-year performance. (**Upper panels**): Bar chart reporting the performance of the five investment strategies over the preceding 10 years in terms of annualized rate of return (**first panel**) and corresponding Sharpe ratio (**second panel**). The (**lower four panels**) report (i) the annualized rate of return relative to a baseline (i.e., buy and hold) strategy, (ii) the principal time constant afforded by modelling the density dynamics illustrated in Figure 4, (iii) the evidence lower bound (ELBO) afforded by the variational free energy of the Helmholtz–Laplace model, and (iv) the evidence for enslaving of assets states, based upon the Bayesian model reduction in (11).

Figure 9 reports a particular example in which we precluded the enslaving of states by themselves to avoid numerical overflow in the long-term projections in Figure 4 and Figure 5. To assess the relative performance of the five strategies, the above analysis was repeated for 10 successive years, from 2025 back to 2016. In other words, working backwards from 2025, the model was inverted using the preceding 256 weeks and used to furnish *ex ante* predictions for the year in question. This means each year of simulated investment was informed by the preceding five years of market fluctuations. The resulting comparative analysis is shown in Figure 10 and reported in tabular format in terms of average (Table 3), best (Table 4) and worst performance (Table 5) over the 10-year period. The best and worst performances are the minimum (or maximum) over the 10 years. This means that the best (and worst) performance on one metric is not necessarily during the same year as the best (and worst) performance on another.

The 10-year average performance was remarkably consistent with the annual performance above: the active inference investor equipped with *ex ante* predictions outperformed other strategies, with a 40% improvement of the rate of return over the buy and hold strategy. Interestingly, the buy and hold strategy was also better than the expected utility investor using *ex post* predictions. The active inference investor with *ex ante* predictions also featured the best performance over the 10 years (of 28.24% in 2021) and the least bad rate of return (of 1.55% in 2018). This reflects the fact that the *ex ante* KL strategy was the only investment strategy that did not lose wealth. However, this does not imply that this strategy consistently outperformed the others, as can be seen in Figure 10.

Figure 10 shows the annualized rate of return in percentage for each of the 10 years for the five investments strategies. The accompanying Sharpe ratios—assessed in terms of month-to-month volatility—are shown in the second panel. These simulation results illustrate the high degree of variability in performance from year to year. In particular, 2018 was a difficult year for all investors. Global stocks suffered their worst quarterly fall towards the end of 2018 amid global economic concerns, driven largely by a trade war between the US and China. The only investor not to lose money was the *ex ante* KL investor. However, there were two years (2023 and 2017) in which this investor was outperformed by the baseline buy and hold strategy. The worst relative performance was seen in 2017.

In 2017, both *ex ante* strategies performed badly in relation to the corresponding *ex post* strategies. This suggests the predictive validity of the Helmholtz–Laplace model is poor, which speaks to an important point. Generally speaking, the posterior predictive accuracy—and consequent predictive validity—go hand-in-hand with model evidence [111]. Crucially, the log evidence for 2017 was the smallest across the 10 years considered. This suggests that the model *per se* was a relatively poor explanation for the data over the preceding five years. This period included the 2015–2016 stock market selloff, when the value of stock prices fell markedly due to successive exogenous shocks, ending with the United Kingdom’s vote to leave the European union on June 9, 2016. In summary, the simulated rate of return can be read as a measure of posterior predictive accuracy, i.e., predictive validity, which converges to log evidence in the limit of large numbers [82,111]. This implies it is necessary—but not sufficient—to base predictions on a model with a high evidence or marginal likelihood.

The lower panels of Figure 10 report some interesting metrics pertaining to the quality of the model for each successive year. The lower panels show the variational free energy or evidence lower bound for the model *per se* and the relative log evidence for enslaving of assets. The third panel reports the performance of different strategies in relation to the buy and hold baseline. Despite the variation in relative performance, the principal (negative) Lyapunov exponent was remarkably consistent (fourth panel), indicating that the horizon of predictability—of the most dissipative modes—is three to four weeks.

## 9. Discussion

The aim of this technical paper was to introduce some procedures that could be applied to financial and other complex system modelling. These procedures include variational treatments of the statistical physics of nonequilibrium steady states [58,60,69], variational Laplace in the service of modelling empirical data [28,75,112], Laplacian approximations to density dynamics [32] and the variational principle of least action in active inference [20,41,43]. Each of these domains constitutes a research field in its own right; however, all of these procedures are drawn from standard techniques in the broader field of complex system modelling. This kind of modelling is sometimes referred to as dynamic causal modelling [24,32,79,113] because it is predicated on a causal model of dynamics inherent in a generative model of how states cause their dynamics.

Many of the procedures described above can be found in the dynamic causal modelling literature, ranging from applications in neuroscience [114,115] through to epidemiology [116,117]. Despite their formal diversity, all of these procedures rest upon the same variational principle of least action enshrined in the Jensen–Feynman inequality. This is an integral part of the free energy principle that can be regarded as dual to Jaynes’ maximum entropy principle, under constraints [101,118]. Another common thread is the use of the Laplace approximation that can be read as truncating some polynomial expansion to second order, as in the application of variational Laplace or the implicit method-of-moments in solving for density dynamics. The particular contribution of the current modelling is to provide a universal functional (Helmholtz–Laplace) form for generative models that can be applied to complex dynamical systems that possess a nonequilibrium steady-state solution. However, committing to this universal functional form comes at the expense of explainability of a certain kind.

Throughout this paper, we have not referred to the meaning or nature of any indicator variable or assets used to illustrate the approach. This means there is a lost opportunity for face-validation, with, perhaps, the exception of Figure 4 (which shows that the model can detect the surprisal associated with historical shocks to the Market). One extension to the modelling would be to equip the hidden states with a narrative or semantics. This is a device that has been considered in computational approaches to medical diagnosis [119]. In brief, the labelling that a portfolio manager or investor would bring to the table—to describe their classification of various states of affairs—can be treated as an observation that, in principle, can be generated and predicted by the evolution of unlabeled hidden states. This would allow regimes of hidden state-space to be labelled in a way that could be interpreted given the diagnostic ontology found in an understanding of financial markets.

This is one of several avenues one might consider. Others include the use of Bayesian model reduction to simplify the model before evaluating the predictive densities required for portfolio management. Another extension would be to cast the state-space model into generalized coordinates of motion [91,120]. This allows one to accommodate serial correlations among the random (exogenous) fluctuations. This speaks to another refinement of the basic procedures above, namely to re-estimate the model by relaxing assumptions about the invertible and deterministic mapping between observable data and hidden states. This is relatively straightforward to implement using generalized or variational filtering that accommodates both state noise—i.e., random fluctuations on the flow of hidden states—and observation noise—i.e., a probabilistic mapping from hidden states to observable data. This would also enable one to explore different parameterizations of the observer function using Bayesian model comparison.

If one wanted to predict the response of the Market to a particular intervention or event, it would be necessary to set the initial conditions in a way that reflected the impact of such an intervention. This requires a mapping from a particular kind of intervention or exogenous shock to the indicator states. In the foregoing, we used the first three principal eigenvariates of all data at hand (including the assets). These are effectively linear mixtures of data, much in the spirit of factor analysis and principal component analysis. However, these mixtures are difficult to label because they summarize many underlying indicator variables that may have a particular meaning for analysts. We therefore return to the limitation of this kind of modelling in terms of its explainability: a price paid for appealing to the constraints afforded by the Helmholtz–Hodge decomposition. In principle, these constraints could be relaxed by considering higher dimensional systems. For example, one could generate all available indicator variables from a small number of hidden states using generalized filtering. One could then use Bayesian model comparison to identify the underlying number or dimensionality of hidden states necessary to explain labelled indicator variables. Similar arguments can be applied to the granularity of aggregated assets or ETFs, speaking to important questions about course graining and scale.

The connection between this work and the mean field game theory could be further strengthened. Mean field game theory describes the average decision making of individual agents within a large population. Agents’ decisions are taken optimal with respect to the time integral of a desired cost function, calling on the Bellman optimality principle and optimal control. Under the free energy principle, optimal control arises as agents minimize their expected free energy, i.e., the time integral of expected free energy. Thus, the optimal policy arising from a specific utility function under Bellman’s equations is equivalently described by an active inference agent acting under an equivalent generative model of its observations. Thus, this work should translate gracefully to mean field game theory and enable studying how distributed belief systems—and discrepancies therein—may influence global market dynamics. Please see [121] for a formal treatment of the relationship between the least action principle, implicit in active inference, and the Bellman optimality principle.

Finally, it is worth speculating on the scale of the approach introduced in this paper. In principle, the modelling and underlying mechanics are scale invariant; c.f., [115,122,123]. This means that one could pick any scale and seek evidence for models based upon a Helmholtz–Laplace decomposition. This could be at the level of milliseconds, as in electrophysiology and seizure prediction, through to decades, as in climate modelling. The particular scale addressed in the current application—namely portfolio management—is largely a question of choice, reflected in the course graining of data used for model selection and inversion. Here, the data were acquired at a scale of days, implying that temporal derivatives or dynamics can only be specified precisely at a scale of a week. This places a lower bound on the temporal scale, which, naturally, leads to forecasting and policy optimization at a scale of months, which will only be manifest on average over a scale of years.

## 10. Conclusions

This paper has focused on the technical details of modelling and policy optimization. However, because it is grounded in the free energy principle, the ensuing description of investors as active inference agents speaks to a larger issue. This turns upon the fact that the free energy principle is just a description of self-organizing systems that possess attracting states. Another way of reading this is that the dynamics of such systems endow them with a persistence or sustainability. On this view, if all investors behaved in accord with the principles of active inference, then one could argue that the free market would not only be sustainable but also resilient to shocks in virtue of minimizing, on average, surprisal and implicitly their entropy. In life sciences, this argument is often cast in terms of a generalized homeostasis (or strictly speaking allostasis), namely sustainable and resilient systems are just those systems that avoid unlikely states with high surprisal. In economics, the same sentiment could be said to underwrite real business cycle (RBC) theory, which sees business cycle fluctuations as an efficient response to exogenous changes in the real economic environment [124]; see also [63] for a deconstruction of RBC theory.

In the current setting, the efficient response is just the path of least action, which—under active inference—is just the path of least risk. In control theory, the same kind of resilience to random fluctuations is underwritten by risk-sensitive or path integral control. One might speculate that the same kind of sustainability is already evident in—or could be engineered by—a commitment to the variational principles of least action. At its simplest, this can be expressed as saying (almost tautologically) that any sustainable system follows the most likely paths, given the kind of system it is.

## Figures and Tables

**Table 1:** (**a**): Exchange-traded funds (ETFs). (**b**): Macroeconomic and financial indicators (FRED tickers).

(**a**)	
SPY	SPDR S&P 500 ETF, representing the U.S. large cap equity market.
VEA	Vanguard FTSE Developed Markets ETF, representing internationally developed equities.
AGG	iShares Core U.S. Aggregate Bond ETF, representing the U.S. investment grade bond market.
VNQ	Vanguard Real Estate ETF, representing U.S. real estate equities.
DBC	Invesco DB Commodity Index Tracking Fund, representing commodity markets.
BIL	SPDR Bloomberg 1–3 Month Treasury Bill ETF, representing short term U.S. government securities.
(**b**)	
GDPC1—Real Gross Domestic Product, quarterly, chained 2012 dollars.	
INDPRO—Industrial Production Index.	
PAYEMS—All Employees, Total Nonfarm.	
UNRATE—Civilian Unemployment Rate.	
ICSA—Initial Claims for Unemployment Insurance.	
TCU—Total Capacity Utilization.	
USSLIND—U.S. Total Business Sales (short-term indicator of industrial activity).	
CPIAUCSL—Consumer Price Index for All Urban Consumers (all items).	
CPILFESL—Core CPI (excluding food and energy).	
PCE—Personal Consumption Expenditures (nominal).	
PCEPILFE—Core PCE Price Index (excluding food and energy).	
PPIACO—Producer Price Index for All Commodities.	
FEDFUNDS—Effective Federal Funds Rate.	
WALCL—Federal Reserve Total Assets (from the balance sheet).	
M2SL—M2 Money Stock.	
TOTALSL—Total Loans and Leases of Commercial Banks.	
BUSLOANS—Commercial and Industrial Loans at All Commercial Banks.	
TB3MS—3-Month Treasury Bill: Secondary Market Rate.	
GS2, GS10, GS30—Constant Maturity U.S. Treasury Yields (2-, 10-, 30-year).	
T10Y2Y—10-Year Treasury Constant Maturity Minus 2-Year Yield (term spread).	
BAA10Y—Moody’s Seasoned Baa Corporate Bond Yield.	
BAA—Moody’s Seasoned Baa Corporate Bond Yield (general).	
BAMLH0A0HYM2—ICE BofA U.S. High Yield Index Option-Adjusted Spread.	
VIXCLS—CBOE Volatility Index (VIX).	
STLFSI2—St. Louis Fed Financial Stress Index.	
CSUSHPINSA—CoreLogic/Case-Shiller U.S. National Home Price Index.	
HOUST—Housing Starts: Total: New Privately Owned Units Started.	
PERMIT—Building Permits: Total New Privately Owned Units.	
MORTGAGE30US—30-Year Fixed Rate Mortgage Average in the U.S.	
DTWEXBGS—Trade Weighted U.S. Dollar Index: Broad, Goods.	
DCOILWTICO—Crude Oil Prices: West Texas Intermediate (WTI).	
PCOPPUSDM—Copper Prices in U.S. Dollars per Metric Ton.	

**Table 2:** Annual performance for 2025.

Strategy	Annual RoR (%)	Volatility (%)	Sharpe Ratio	Drawdown (%)
*buy & hold*	8.65	26.8	0.24	−4.7
*ex post* EU	7.10	30.6	0.16	−6.0
*ex post* KL	10.38	36.0	0.23	−7.0
*ex ante* EU	11.32	39.8	0.23	−6.8
*ex ante* KL	**11.63**	38.3	**0.25**	−7.0

**Table 3:** Annual performance: 10-year average.

	Annual RoR (%)	Volatility (%)	Sharpe Ratio	Drawdown (%)
*buy & hold*	7.13	30.6	**0.28**	−3.7
*ex post* EU	6.72	37.4	0.20	−4.1
*ex post* KL	8.10	41.0	0.22	−4.7
*ex ante* EU	9.42	35.5	0.26	−4.2
*ex ante* KL	**9.98**	37.2	**0.28**	−4.3

**Table 4:** Annual performance: 10-year best.

	Annual RoR (%)	Volatility (%)	Sharpe Ratio	Drawdown (%)
*buy & hold*	19.73	11.1	**1.11**	−1.6
*ex post* EU	27.77	13.8	0.89	−1.5
*ex post* KL	27.66	22.2	0.85	−2.1
*ex ante* EU	26.50	7.7	1.07	−0.7
*ex ante* KL	**28.24**	8.6	1.10	−0.9

**Table 5:** Annual performance: 10-year worst.

	Annual RoR (%)	Volatility (%)	Sharpe Ratio	Drawdown (%)
*buy & hold*	−6.25	79.3	−0.18	−10.8
*ex post* EU	−4.66	79.7	−0.15	−9.6
*ex post* KL	−7.68	85.9	−0.15	−12.5
*ex ante* EU	−1.53	79.7	−0.13	−8.5
*ex ante* KL	**1.55**	89.6	**−0.02**	−9.9

## Data Availability

The MATLAB (Version R2025b) code associated with this manuscript is available in the DEM toolbox of the open-source SPM software (development version, https://github.com/spm/spm) (accessed on 16 June 2026). Equity and commodity prices were sourced from Yahoo Finance using the yfinance API (https://ranaroussi.github.io/yfinance/reference/index.html accessed on 16 June 2026). Macroeconomic and financial series were obtained from the Federal Reserve Economic Data (FRED) database via pandas_datareader (https://pandas-datareader.readthedocs.io/en/latest/ (accessed on 16 June 2026)).
